# Loss of *DPP6* in neurodegenerative dementia: a genetic player in the dysfunction of neuronal excitability

**DOI:** 10.1007/s00401-019-01976-3

**Published:** 2019-03-14

**Authors:** Rita Cacace, Bavo Heeman, Sara Van Mossevelde, Arne De Roeck, Julie Hoogmartens, Peter De Rijk, Helena Gossye, Kristof De Vos, Wouter De Coster, Mojca Strazisar, Greet De Baets, Joost Schymkowitz, Frederic Rousseau, Nathalie Geerts, Tim De Pooter, Karin Peeters, Anne Sieben, Jean-Jacques Martin, Sebastiaan Engelborghs, Eric Salmon, Patrick Santens, Rik Vandenberghe, Patrick Cras, Peter P. De Deyn, John C. van Swieten, Cornelia M. van Duijn, Julie van der Zee, Kristel Sleegers, Christine Van Broeckhoven, the BELNEU Consortium, Johan Goeman, Roeland Crols, Dirk Nuytten, Jan L. De Bleecker, Tim Van Langenhove, Adrian Ivanoiu, Olivier Deryck, Bruno Bergmans, Jan Versijpt, Alex Michotte, Jean Delbeck, Christiana Willems, Nina De Klippel

**Affiliations:** 1 Center for Molecular Neurology, VIB, Antwerp, Belgium; 2 Institute Born-Bunge, Antwerp, Belgium; 3 University of Antwerp, Antwerp, Belgium; 4 Department of Neurology, Antwerp University Hospital, Edegem, Belgium; 5 Department of Neurology and Memory Clinic, Hospital Network Antwerp (ZNA), Middelheim and Hoge Beuken, Antwerp, Belgium; 6 Switch Laboratory, VIB-KU Leuven Centre for Brain and Disease Research, Louvain, Belgium; 7 Switch Laboratory, Department of Cellular and Molecular Medicine, KU Leuven, Louvain, Belgium; 8 Department of Neurology, University Hospital Ghent and University of Ghent, Ghent, Belgium; 9 Department of Neurology, Centre Hospitalier Universitaire de Liège and University of Liège, Liège, Belgium; 10 Department of Neurosciences, Faculty of Medicine, KU Leuven, Louvain, Belgium; 11 Laboratory of Cognitive Neurology, Department of Neurology, University Hospitals Leuven, Louvain, Belgium; 12 Department of Neurology, Erasmus University Medical Centre, Rotterdam, The Netherlands; 13 Department of Epidemiology, Erasmus University Medical Centre, Rotterdam, The Netherlands; 14 Neurodegenerative Brain Diseases Group, VIB Center for Molecular Neurology, University of Antwerp, CDE, Universiteitsplein 1, 2610 Antwerp, Belgium

**Keywords:** Neurodegenerative brain diseases, Dementia, DPP6, Hyperexcitability, Whole genome sequencing, Oxford nanopore technologies (ONT) PromethION

## Abstract

Emerging evidence suggested a converging mechanism in neurodegenerative brain diseases (NBD) involving early neuronal network dysfunctions and alterations in the homeostasis of neuronal firing as culprits of neurodegeneration. In this study, we used paired-end short-read and direct long-read whole genome sequencing to investigate an unresolved autosomal dominant dementia family significantly linked to 7q36. We identified and validated a chromosomal inversion of ca. 4 Mb, segregating on the disease haplotype and disrupting the coding sequence of dipeptidyl-peptidase 6 gene (*DPP6*). *DPP6* resequencing identified significantly more rare variants—nonsense, frameshift, and missense—in early-onset Alzheimer’s disease (EOAD, *p* value = 0.03, OR = 2.21 95% CI 1.05–4.82) and frontotemporal dementia (FTD, *p* = 0.006, OR = 2.59, 95% CI 1.28–5.49) patient cohorts. DPP6 is a type II transmembrane protein with a highly structured extracellular domain and is mainly expressed in brain, where it binds to the potassium channel K_v_4.2 enhancing its expression, regulating its gating properties and controlling the dendritic excitability of hippocampal neurons. Using in vitro modeling, we showed that the missense variants found in patients destabilize DPP6 and reduce its membrane expression (*p* < 0.001 and *p* < 0.0001) leading to a loss of protein. Reduced DPP6 and/or K_v_4.2 expression was also detected in brain tissue of missense variant carriers. Loss of DPP6 is known to cause neuronal hyperexcitability and behavioral alterations in Dpp6-KO mice. Taken together, the results of our genomic, genetic, expression and modeling analyses, provided direct evidence supporting the involvement of DPP6 loss in dementia. We propose that loss of function variants have a higher penetrance and disease impact, whereas the missense variants have a variable risk contribution to disease that can vary from high to low penetrance. Our findings of *DPP6*, as novel gene in dementia, strengthen the involvement of neuronal hyperexcitability and alteration in the homeostasis of neuronal firing as a disease mechanism to further investigate.

**Electronic supplementary material:**

The online version of this article (10.1007/s00401-019-01976-3) contains supplementary material, which is available to authorized users.

## Background

Considerable progress has been made towards understanding the genetic origin, the neuropathology and the epidemiology of neurodegenerative brain diseases (NBD) such as Alzheimer’s disease (AD) and frontotemporal dementia (FTD). Linkage analyses and large-scale genomics studies, have shown that a wide genetic heterogeneity is responsible for the neuronal pathologies and dysfunctions in NBD [6, 34, 61], with yet additional causes to identify and mechanisms to discover. Many disorders of the central nervous system (CNS) show a broad array of clinical features, e.g., impaired behavior, language, etc., but some of them, such as alterations in memory, are shared across disorders [7, 28, 55]. Several emerging concepts suggest a converging mechanism in NBD involving early neuronal network dysfunctions [58, 84] and alterations in the homeostasis of neuronal firing [24] as culprits of neurodegeneration [24, 58, 84]. This is supported by studies in mouse models of AD, in which was shown that higher firing rates can promote amyloid-β (Aβ) production [84] and that the neuronal hyperactivity precedes the deposition of plaques [5]. Studies using functional magnetic resonance (fMRI) demonstrated hippocampal activation in both patients with amnestic mild cognitive impairment (aMCI) [16] and preclinical carriers of inherited causal mutations in familial AD [64] as well as FTD patients [83]. These are only some examples of an emerging field, which spurs the investigation of these new mechanisms to better understand neurodegenerative dementia and to design more effective treatments [3, 58]. Despite the diverse mechanistic insights [24, 58, 84], additional data are necessary to better understand the pathophysiology of the early dysfunctions of circuits and neurons. Our discoveries, started with an in depth examination of an unexplained linked locus on chromosome 7q36 (size 5.44 Mb and LOD score = 3.39 at *θ* = 0) [65] and resulted in the finding of dipeptidyl-peptidase 6 (*DPP6*) as novel gene in NBD. DPP6 is a single pass type II transmembrane protein expressed in brain, where it forms a multimeric complex with the potassium channel K_v_4.2, regulating the voltage-dependent gating properties and the surface expression of K_v_4.2 in the brain [60] playing a crucial role in neuronal excitability [73]. With this study we provided direct genetic evidence to support the involvement of neuronal hyperexcitability and alteration in the homeostasis of neuronal firing as disease mechanisms in dementia.

## Materials and methods

### Family 1270

The proband of family 1270, aged 47 years, was ascertained in the frame of the Dutch population-based epidemiological study of early-onset AD in 1980–1987 [30]. The 1270 family, with a history of autosomal dominant inheritance [29, 76], was sampled for genetic studies in the 80–90’s. Diagnoses of AD were made according to National Institute of Neurological and Communicative Diseases and Stroke–Alzheimer’s Disease and Related Disorders Association (NINCDS-ADRDA) criteria published in 1984 [49], and of mild cognitive impairment (MCI) according to Honig and Mayeux [31]. Linkage analyses for loci on chromosomes 14, 19, and 21 [76], and mutation screening of the AD genes, *APP* (21.q21.1), *PSEN1* (14q24.3) and *PSEN2* (1q42.1), were negative [11, 75].

Follow-up clinical studies of family 1270, including neurological examination of incident patients, interviews of first-degree relatives and review of medical records and CT scans, identified four additional patients [65]. The onset age in the updated family was highly variable with a mean age at onset of 66.8 ± 7.4 years and range of 47–77 years. In the proband, and most other patients, the disease initially presented with memory impairment, except for one patient, in whom a change of personality was the initial complaint, which later in the disease course was followed by memory loss. In all patients the disease progressed into other areas of cognition, such as praxis and speech [65]. Neuroimaging was available for two patients, III-48 at age 74 years and III-21 at age 82 years and showed cortical atrophy in both [65]. For the only living patient, III-21, who received the diagnosis of possible AD, a CT scan (at age 82 years) showed that cortical and subcortical atrophy was most notable in the temporal and frontal regions (supplementary material) [65]. A cohort of 82 Dutch EOAD patients [76], mean age at onset [AAO] ± standard deviation (SD) of 57.0 ± 5.6 years (82.3% women) was included for candidate gene resequencing.

### Belgian patient and control cohorts

The Belgian AD cohort consisted of 558 patients (mean onset age 61.6 ± 6.8 years, range 33–70 years), of whom 221 Belgian AD patients were referred to our molecular diagnostic unit for *PSEN1*, *PSEN2*, and/or *APP* mutation screening. Post-mortem brain analysis was performed in 17 patients confirming AD pathology. The majority of the patients were recruited at the memory clinic of the hospitals Middelheim and Hoge Beuken of the Hospital Network Antwerp (ZNA), Belgium (P.P.D.D. and S.E.) [19, 20]. Another subset was collected at the Department of Neurology and the Memory Clinic of the University Hospitals of Leuven (UHL), Belgium (R.V.) as well as through the neurology centers of the clinical partners of the Belgian Neurology (BELNEU) consortium. Diagnosis of possible, probable or definite AD was obtained by consensus of at least two neurologists based on the NINCDS-ADRDA diagnostic criteria [49] and the National Institute on Aging-Alzheimer’s Association (NIA-AA) diagnostic criteria [32, 50]. Each AD patient underwent a neuropsychological examination, including mini-mental state examination (MMSE) [23] and structural neuroimaging, while functional neuroimaging and cerebrospinal fluid analysis was done in a subset of patients [4]. The Belgian FTD cohort consisted of 614 patients (mean AAO 66.1 ± 9.9 years; age range 20–89 years), which included 35 patients with a concomitant amyotrophic lateral sclerosis (FTD-ALS), recruited in the framework of the BELNEU consortium [25, 78]. Clinical FTD diagnosis was made according to established clinical criteria [54, 66]. Post-mortem pathological analysis confirmed diagnosis in 29 FTD and 3 FTD-ALS patients. In the screened cohort, a total of 100 patients carried a mutation in a known causal dementia gene: 51 patients (8.3%) carried a *C9orf72* pathogenic repeat expansion, 31 patients (5%) had a pathological *GRN* mutation, five patients (0.8%) carried a mutation in *TBK1*, four patients (0.6%) had a mutation in *MAPT*, six patients (1%) had a *VCP* mutation, one patient had a *CHMP2B* mutation (0.2%), a *TARDBP* mutation (0.2%) or a *PSEN1* mutation (0.2%). The Belgian control cohort consisted of 755 unrelated and non-demented individuals [mean age at inclusion (AAI), 71.6 ± 9.7 years; age range 34–100 years]. In the selection of control persons, subjective memory complaints and neurological or psychiatric antecedents, as well as a familial history of neurodegeneration, were ruled out by means of an interview. Cognitive screening was initially performed using the mini-mental state examination (MMSE, cutoff > 25) [23] but later the Montreal Cognitive Assessment (MoCA, cutoff > 25) [53] was also used. The majority of the control persons were community-dwelling volunteers. Additionally spouses of patients were included after examination at the Memory Clinic of the ZNA Middelheim and Hoge Beuken hospitals, Antwerp, Belgium and the Memory Clinic of the University Hospitals Leuven, Gasthuisberg, Leuven, Belgium.

### Ethical assurances

Ascertainment of the family 1270 relatives and the Dutch patients was performed in the Netherlands using a study protocol approved by the Medical Ethical Committee of the Erasmus Medical Center Rotterdam. All Belgian participants and/or their legal guardian signed a written informed consent form for their participation in the clinical and genetic studies. The clinical and genetic study protocols and the informed consent forms were approved by the Ethics Committee of the University Hospital Antwerp and the University of Antwerp, and the respective hospitals of the members of the BELNEU consortium, Belgium.

### Whole genome sequencing (WGS)

Short-read paired-ends WGS was performed at Complete Genomics Inc. (Mountain View, CA USA) [18]. Raw sequencing reads were aligned to the reference genome (National Center for Biotechnology Information (NCBI) build 36 (hg18). Sequence alignment and variant calling were performed by Complete Genomics Inc. while data annotation and analysis were performed with the GenomeComb package [68]. Good quality variants were selected as previously described [68]. Additionally, novel or rare (minor allele frequency (MAF) < 1% in the 1000 Genome Project [2] and/or in our in-house database of WGS data of unrelated individuals (n = 82)), heterozygous variants shared among the four WGS patients were investigated. Sequenom MassARRAY^®^ (Agena Bioscience, CA, USA) and Sanger sequencing (BigDye Terminator Cycle Sequencing kit v3.1; analysis on an ABI 3730 DNA Analyzer, both Thermo Fisher Scientific, MA, USA) were used for variants validation. Structural variations (SV) were called by Complete Genomics and/or using a SV detection tool integrated in GenomeComb [68] version 0.90.0 (available at http://genomecomb.sourceforge.net). This tool scans the genome sequencing data for groups of read pairs that map at a distance that is markedly different from the expected insert size. Under normal conditions, the distance between the two sequence reads of a mate pair is expected to be approximately 350 bp corresponding to the size of selected fragments during WGS library construction. For the detection of inversions, groups of discordant mate pairs must be present for which the reads map at a different distance than expected and in opposite orientation. The linkage region between markers D7S636 and D7S559 [65] was analyzed for both single nucleotide variants (SNVs) as well as SV. Additionally, the public genome data repository of Complete Genomics Inc. containing freely accessible WGS data of 69 individuals, 52 unrelated individuals (software version 1.10.0; http://www.completegenomics.com/public-data/) [18], as well as WGS data from 427 individuals (157 unrelated) sequenced by Complete Genomics Inc. (software version 2.2.0) and distributed by the 1000 genome project [2] were used for comparative purposes.

Long-read direct WGS was performed in-house on the PromethION sequencing platform (Oxford Nanopore Technologies (ONT), UK). Prior to library preparation, the DNA was sheared to 35 kb using the Megaruptor (Diagenode, BE) and size selected to a minimal length of 6 kb on the BluePippin (Sage Science, MA, USA) using a High-pass protocol and the S1 external marker on a 0.75% agarose gel (Sage Science, USA). The recommended SQK-LSK109 protocol for library preparation for PromethION (ONT) sequencing was followed with slight increases in all enzymatic incubation times and during elution. In short, DNA template damage and ends were repaired in a combined step using NEBNext FFPE DNA Repair Mix and NEBNext Ultra II ER/dAT Module (New England Biolabs, USA) followed by AMPureXP (Beckman Coulter, CA, USA) bead purification and ligation of platform-specific adapter sequences. The final library (100 fmol) was loaded on a PromethION flow cell with 8021 active pores at the start, following the default protocol for PromethION DNA sequencing. Base calling of the raw reads was performed using the ONT basecaller Guppy (v1.4.0) on the PromethION compute device. Run metrics were calculated and visualized using NanoPack [12]. Reads were aligned to hg19 using ngmlr (v0.2.6) [70] using default parameters. Inversions were detected using npInv inversion caller [71]. The coverage was assessed using mosdepth [59]. Long-read WGS of ten unrelated individuals (eight dementia patients and two controls) generated in-house following the same pipeline was used for comparison purposes.

DNA local alignment analysis of the NCBI hg19 reference sequence of chromosome 7: 149,169,800–154,794,690 bp was performed using YASS [57].

### Directional genomic hybridization (dGH™) analysis

Directional genomic hybridization (dGH™) [67] analysis of chromosome 7 was performed by KromaTiD, Inc. (Fort Collins, Colorado, http://www.kromatid.com/) using the dGH™ C7 Paint assay (D3P-HC710) on Epstein–Barr virus (EBV) immortalized lymphoblast cell lines fixed in metaphase after one replication cycle in the presence of Brd-U and Brd-C. Single-stranded sister chromatids were hybridized with high density directional probes. Inverted fragments inherently possess an opposite 5′ → 3′ orientation, resulting in a switch of fluorescent hybridization signal from one sister chromatid to the other.

### Whole gene resequencing

Resequencing of the complete coding region (CDS) of *DPP6* (NM_130797.3), including two alternatively spliced exon 1 (NM_001936.4 and NM_001039350.2) and intron/exon boundaries, was achieved using a custom-designed gene panel (Agilent Technologies, CA, USA) [26] combined with massive parallel sequencing (MPS) on a MiSeq^®^ sequencer (Illumina^®^, San Diego, CA, USA). Read processing, alignment and variant calling were performed in-house with a standardized pipeline integrated in GenomeComb [68]. The pipeline used fastq-mcf [1] for adapter clipping. Reads were then aligned using bwa [41]. Realignment in the neighborhood of indels was performed with GATK [15]. All positions with a coverage ≥ 5 were variant called using GATK [15]. At this initial stage positions with a coverage < 5 or a score < 30 were considered unsequenced. The resulting variant sets of different individuals were combined and annotated using GenomeComb [68]. Downstream data analysis was further performed with the same software [68]. Exon 1 of DPP6 isoform 1 (NM_130797.3), due to high GC content and genomic complexity, was sequenced upon PCR amplification with specific primers. PCR products were processed by direct Sanger sequencing as described earlier in the text. Sanger sequencing was also used for validation of the identified variants after gene panel sequencing assay, using exon-specific primer pairs (sequences available upon request).

### Variants modeling

Prediction of deleteriousness of the nucleotide changes for the *DPP6* variants was performed using Combined Annotation Dependent Depletion (CADD) version 1.3. The rescaled (PHRED) score is reported, which correlates with allelic diversity and variants pathogenicity [37]. The investigation of the effect of the amino acid changes on protein stability (difference in free Gibbs energy) and the interaction with functional residues (i.e., glycosylation sites) were performed with FoldX (http://foldx.crg.es/) and YASARA [39, 77]. This analysis was limited to the extracellular domain, because only the crystallographic structure of this protein domain is available. DPP6 protein data bank accession number: 1XFD.

### DPP6 transmembrane protein stability assay

Gateway and In-Fusion cloning (both Invitrogen, Thermo Fisher Scientific, Waltham MA, USA) were used to generate the wild-type DPP6 pCR3 expression construct C-terminally fused with the HiBit sequence as well as a control construct including a PEST sequence in between DPP6 and the HiBit sequence (constructs available upon request). Mutations of interest were introduced in the wild-type DPP6 construct by site directed in vitro mutagenesis using KAPA HiFi HotStart DNA polymerase (Kapa Biosystems, MA, USA). A construct containing the sequence of the secreted Gaussia luciferase (GLuc) was used for normalization purposes. HEK293T cells were co-transfected with DPP6 and GLuc constructs (4:1 ratio) using XtremeGene9 (Sigma-Aldrich, MO, USA). Non-transfected cells were included as a control. Gaussia luciferase and Nano-Glo^®^ HiBit luciferase signals were detected 48 h after transfection by the use of BioLux Gaussia Luciferase Assay Kit (New England Biolabs, MA, USA) and a Nano-Glo^®^ HiBit Extracellular detection System (Promega Corporation, WI, USA), respectively. Both the GLuc and the Nano-Glo^®^ HiBiT luminescence signal (LUC) were measured following the manufacturer guidelines using a GloMax^®^96 microplate luminometer (Promega Corporation, WI, USA). The injector option was used for detection of GLuc activity. All construct concentrations and LUC signals were initially optimized to be within the linear range of detection. For data analysis, relative LUC activity was calculated as Nano-Glo^®^ HiBiT luciferase signals normalized to GLuc signals. Six independent experiments were performed and the resulting data per construct were pooled together for statistical analysis.

### DPP6 mRNA and protein analyses

Semi-quantitative real-time PCR (qRT-PCR) was used to quantify brain expression levels of total *DPP6*. Expression levels were measured in the frontal cortex (BA10) of patient-specific variant carriers (*n* = 3) and control individuals (*n* = 4). Total RNA was isolated from fresh frozen brain tissue using the RiboPure™ kit followed by DNase treatment with TURBO DNase (both Ambion, Thermo Fisher Scientific, MA, USA). First-strand cDNA was synthetized utilizing the SuperScript^®^ III First-Strand Synthesis System (Thermo Fisher Scientific, MA, USA) with random hexamer primers. qRT-PCR reactions were performed using the Fast SYBR^®^ Green chemistry (Thermo Fisher Scientific, MA, USA) and run on the ViiA™ 7 Real-Time PCR System (Thermo Fisher Scientific, MA, USA). Quantification of mRNA levels was achieved with glyceraldehyde 3-phosphate dehydrogenase (*GAPDH*), tyrosine 3-monooxygenase/tryptophan 5-monooxygenase activation protein, zeta polypeptide (*YWHAZ*), hypoxanthine phosphoribosyltransferase 1 (*HPRT1*), TATA box binding protein (*TBP*) as internal control genes, all with moderate to high expression in neurons (https://www.proteinatlas.org/). Normalization to the reference genes was achieved through geometric averaging of the expression levels, as described by Vandesompele and colleagues [80]. Each sample was measured in triplicate and three independent experiments were performed.

Protein lysates from fresh frozen human brain tissue were made in modified radioimmunoprecipitation (RIPA) buffer (150 mM NaCl, 0.5% sodium deoxycholate, 1% NP-40, 50 mM Tris–HCl; pH 8.0) supplemented with 1% sodium dodecyl sulfate (SDS), as described previously [38]. Protein preparations from mutation carriers and control individuals were separated on 4–12% NuPAGE^®^ Bis–Tris gel (Thermo Fisher Scientific, MA, USA) and electroblotted onto a polyvinylidene difluoride membrane (PVDF, Hybond P; Amersham Biosciences, GE Healthcare Life Sciences, Buckinghamshire, UK). Membranes were probed with primary antibodies to detect DPP6 (1:10,000 AVIVA System Biology, ARP44867_P050, San Diego, CA, USA), K_v_4.2 (1:1000 Abcam, (EP982Y) ab46797, Cambridge, UK) and GAPDH (1:20,000 GeneTex, GTX100118, Irvine, CA, USA). Immunodetection was performed with specific secondary antibodies conjugated with horseradish peroxidase (HRP) and the ECL-plus chemiluminescent detection system (GE Healthcare Life Sciences). Western blot results were visualized and quantified using the ImageQuant™ LAS4000 digital imaging system and the ImageQuant™ TL software (GE Healthcare Life Sciences, Buckinghamshire, UK). Independent protein preparations and western blot experiments were performed three times.

### Statistical analyses

For the rare variants analysis, power calculation was performed within the SKAT framework in R (version 3.1.2). A logistic test for dichotomous traits with a SKAT-O model was used (target sequence: 3545 bp, causal variant percentage = 40%, protective variant percentage = 10%, Maximal OR = 5). Under these conditions both patient-control cohorts (> 1300 individuals) reached the power requirement level of 80% with a 0.05 significance level, since the total sample cohort required for this level is at least 925 individuals. Gene-based burden analysis of rare variants (minor allele frequency (MAF) < 1%) was performed with SKAT-O in R, version 3.1.2, using the SKAT package. Adjustment was applied because the sample size was < 2000. A two-sided *p* value < 0.05 was considered significant. Odds ratio (OR) and 95% confidence interval (CI) were calculated using an allelic Fisher’s exact test. To investigate the effect of *DPP6* rare variants on AAO, disease duration (DD) and family history (FH), we tested the patients with available records of the specific phenotypical traits. Patients carrying a *DPP6* variant were compared to the non-carrier patients’ cohorts. Patients with a pathogenic mutation in known causal genes were excluded. A two-tailed non-parametric Mann–Whitney *U* test was applied to test for an effect on AAO and DD in GraphPad Prism 6 (La Jolla, CA, USA). A *χ*^2^ test was applied to test for enrichment in familial load in carriers of *DPP6* rare variants compared to non-carrier patients. For the mRNA expression studies, three qRT-PCR experiments were independently normalized as previously described [80] and the results were pooled. The non-parametric two-tailed Mann–Whitney *U* test was used to compare the expression of total DPP6 levels between patients, carriers of *DPP6* variants and control individuals in GraphPad Prism 6 (La Jolla, CA, USA). A two-tailed *p* value < 0.05 was considered significant. Western blot results were quantified using the ImageQuant™ TL software (GE Healthcare Life Sciences, Buckinghamshire, UK). Relative protein expression levels between mutation carriers and control individuals were analyzed using a two-tailed Student’s *t* test. A *p* value < 0.05 was considered significant. For the protein stability assay, the normalized LUC values of six experiments were pooled per variant and compared to the wild-type levels using a non-parametric Kruskal–Wallis test in combination with a post hoc Dunn’s test in GraphPad Prism 6 (La Jolla, CA, USA).

## Results

### Family 1270

**Fig. 1:**
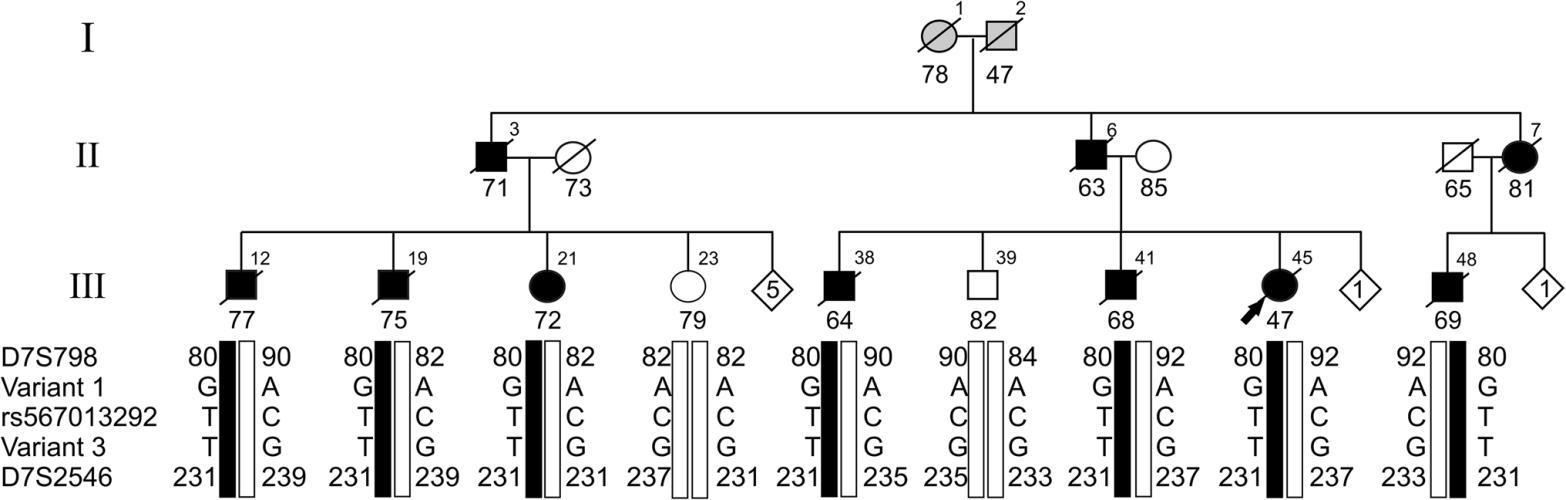
Segregation of *DPP6* in family 1270. Segregation analysis of three rare variants identified in WGS data in intron 1 of *DPP6* (hg18 variant 1 g.153577081 A>G, variant 2 g.153737600 C>T; rs567013292 and variant 3 g.153744958 G>T) delimited by the STR markers D7S798 and D7S2546. Black bars represent the disease haplotype of patients. Numbers within each diamond are unaffected individuals, non-carriers of the disease haplotype included in the genotyping. Arabic numbers above the symbols denote individuals, Arabic numbers below the symbols denote age at onset for patients or either age at last examination or age at death for unaffected individuals. The arrow identifies the proband in the family. WGS data were generated for patients III-12, III-38, III-41, and III-48 from three different sib ships of the pedigree. Direct long-read WGS on Oxford Nanopore PromethION sequencer was performed for III-48. Directional genomic hybridization was performed in cell lines derived from patient III-48 (Fig. 3) and from the non-carriers III-23 and III-39

Earlier, the overall clinical picture of dementia in the mutigenerational family 1270 was reported to be compatible with AD (Fig. 1, Fig. S1) [65, 76]. However, at the time of diagnosis of the index patient and of the affected relatives, cerebrospinal fluid (CSF) biomarkers and amyloid brain imaging were not available. Also, there was no autopsied brain available to obtain a definite diagnosis of dementia subtype based on neuropathology. Previous genetic analyses in the linked locus 7q36 in family 1270 [65] identified seven variants in five different genes: ATP-binding cassette subfamily F, member 2 (*ABCF2*); *N*-acetylgalactosaminyltransferase 11 (*GALNT11*); *DPP6*; PAX transcription activation domain interacting protein (*PAXIP1*) and Engrailed 2 (EN2), which were segregating on the disease haplotype in family 1270 [65]. Most of these variants were synonymous and only the *PAXIP1* p.A660 variant was absent from control individuals [65]. Current public genetic data (e.g., ExAc) [40], showed different nucleotide changes in *PAXIP1*, leading to the same silent variant (p.A660), making it unlikely that the *PAXIP1* variant has a deleterious effect. Further, we found no evidence for aberrant splicing of *PAXIP1* in lymphoblast cells of patients [65].

### WGS analysis of single nucleotide variants in the 7q36 locus

**Fig. 2:**
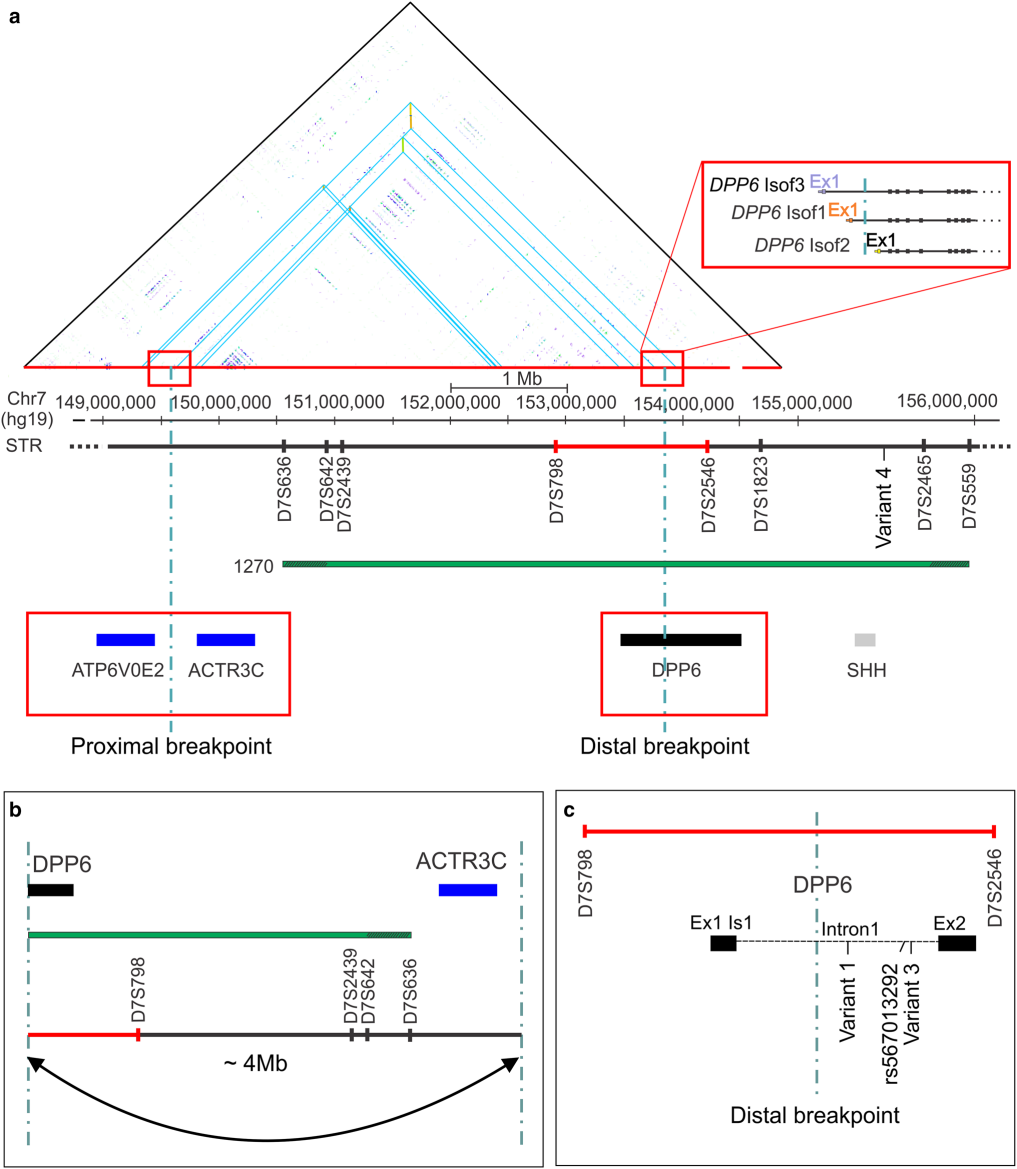
DNA local alignment and schematic representation of 7q36 inversion disrupting *DPP6*. **a** DNA local alignment analysis of the NCBI hg19 reference sequence of chromosome 7: 149,169,800–154,794,690 bp shows inverted low copy repeats (LCRs) indicated by blue triangles. The horizontal green bar represents the candidate region of 5.44 Mb (reference build hg19) linked to 7q36 in family 1270, between the short tandem repeats (STRs) markers D7S636 and D7S559 [65]. The dotted vertical lines mark the locations of the proximal and distal inversion breakpoints, with the distal breakpoint in *DPP6* (black bar) and the proximal breakpoint in the intergenic region between the genes *ATP6V0E2* and *ACTR3C* (blue bars). Red rectangles magnify the location of the proximal and distal breakpoints. The distal breakpoint is located within the intron 1 of *DPP6.* Three isoforms are reported in the figure with independent transcription starting sites and regulatory elements (top right red rectangle). **b** Visualization of the 180° flip of the genomic sequence by the inversion, separating the regulatory region and exon 1 from the coding sequence of DPP6. **c** Magnification of the region around the distal inversion breakpoint in intron 1 of *DPP6* between D7S798 and D7S2546 (red bar) and the location of the three co-segregating rare variants identified by WGS studies. The fourth segregating variant (variant 4) is reported in **a** downstream of the *SHH* gene (grey)

In family 1270 (Fig. S1), we performed paired-end WGS on high molecular weight genomic DNA of four patients in three different sibships of generation III, i.e., patients III-12, III-38, III-41, and III-48 (Fig. 1). On average 95.5% of the genome sequence had reliable diploid calls in all four patients. The genetic variants were annotated and good quality [68], rare or heterozygous non-coding variants (< 1% in 1000 Genome Project) [2], that were shared between the four patients, were further investigated. In the linked locus, between STR markers D7S636 and D7S559 [65], variant selection retained 79 non-coding variants. Validation and segregation analysis in family 1270 retained 38 non-coding variants that co-segregated in family 1270 on the disease haplotype. Genotyping of the variants in control individuals, showed that four of the variants were unique to family 1270 (Fig. 1). Variant 1 (chr7:g.153577081 A>G), variant 2 (chr7:g.153737600 C>T; rs567013292), and variant 3 (chr7:g.153744958 G>T) are all located in intron 1 of *DPP6* (Figs. 1, 2). Variant 4 (chr7:g.155325040 G>C) mapped in an intergenic region, 27.3 kb distal of the closest gene *SHH* (Fig. 2), which is located within the linked locus, 908 kb downstream of *DPP6*. None of the four variants had a high disruptive potential based on the ENCODE [69] annotation.

To exclude that coding variants outside the linked locus were causing the disease, we extracted the exome from the WGS data and analyzed the presence of rare and/or novel coding non-synonymous variants. Heterozygous variants, shared by the four patients, were selected (UTRs, synonymous and non-synonymous), and filtered based on quality [68] and frequency similar as for the non-coding variants. Only the rare/novel (< 1% in 1000 Genome Project) [2] variants in known protein coding genes, that predicted to impact the protein sequence (non-synonymous), were validated. This selection generated nine non-synonymous variants (Table S1), but none co-segregated with disease in family 1270.

### An inversion of 4 Mb disrupting the *DPP6* sequence was detected in the linked 7q36 locus

Investigation of the variants within the linked region evidenced the presence of inverted low copy repeats (LCRs) in the 7q36 locus. In silico simulation of amplification of primers, designed to validate these variants, showed PCR products that aligned in opposite orientations at two loci on chromosome 7, separated from each other by about 4 Mb. Local alignment of the DNA regions between 149,169,800 and 154,794,690 bp on chromosome 7 (hg19), confirmed the presence of inverted paralogous low copy repeats (IP-LCRs) with > 98% sequence homology and located ca. 4 Mb apart (Fig. 2). Bioinformatics analysis of the WGS data of the four patients identified a paracentric (sub-telomeric) inversion in the q-arm of chromosome 7 of about 4 Mb (inv(7)) (Fig. S2) with the inversion breakpoints located within the IP-LCRs regions (Fig. 2). The distal breakpoint is located within the linked locus at 7q36.2, in intron 1 of dipeptidyl-peptidase 6 (*DPP6*), and it is predicted to disrupt the coding sequence of the gene (Fig. 2). The proximal inversion breakpoint is located outside the linked locus, in an intergenic region between proximally *ATP6V0E2* at 122 kb and distally *ACTR3C* at 243 kb (Fig. 2). These results were confirmed by direct long-read WGS on PromethION performed for patient III-48. The sequence run generated 21.2 Gbase of data resulting in a median coverage of 6×. The npInv inversion caller [71] independently identified an inversion at chr7:149,704,610–153,786,893 confirming the previous findings. The inversion at 7q36.2, with one breakpoint in intron 1 of DPP6, was not detected in publicly available WGS data of 209 unrelated individuals, all sequenced with the same short-read sequencing technology. Also, the inversion was not present in long-read WGS of ten unrelated dementia patients and control persons, generated by us using the Oxford Nanopore PromethION sequencer.

### In vitro visualization of the 7q36 inversion

**Fig. 3:**
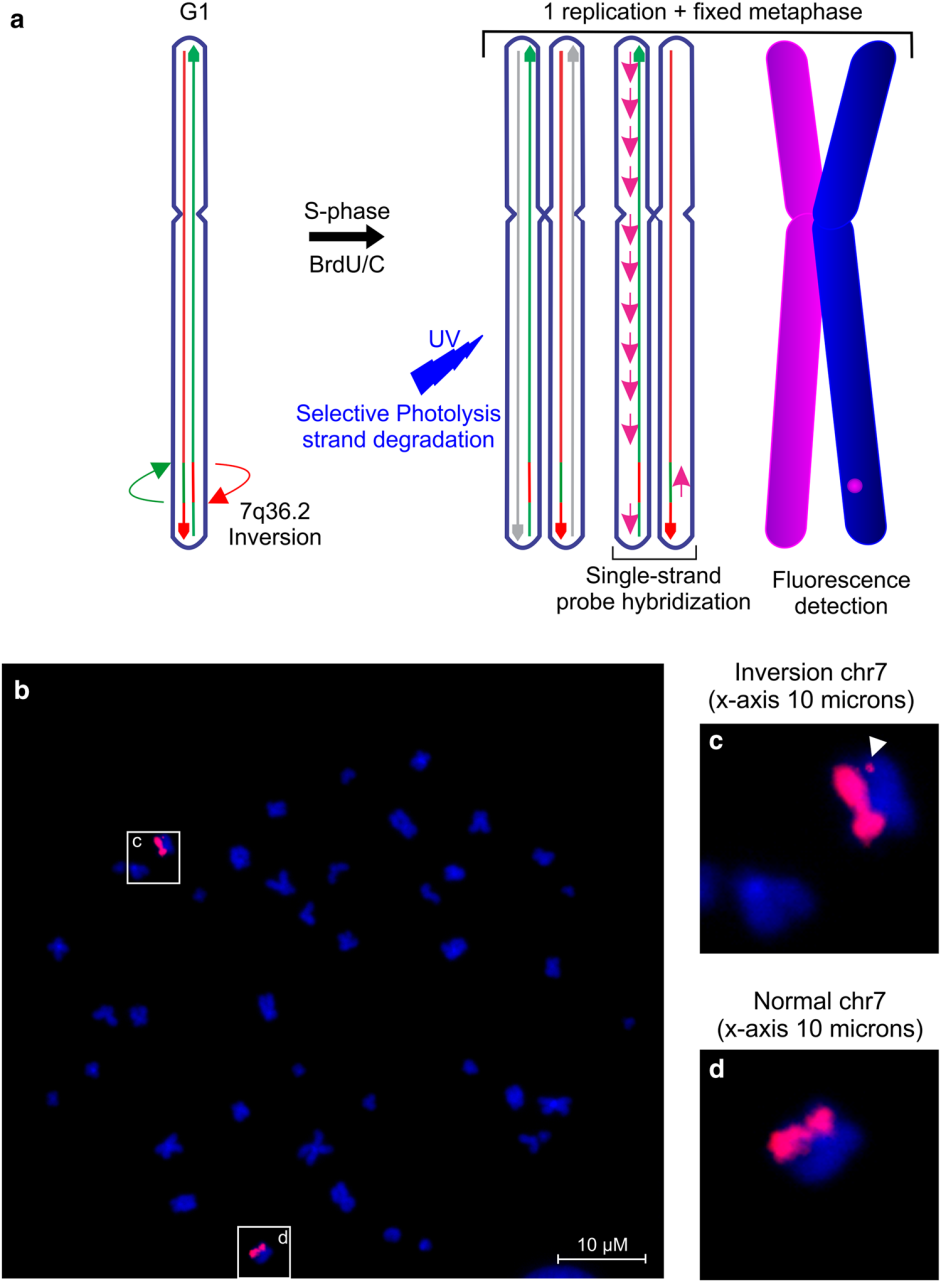
Inversion validation by directional genomic hybridization. **a** Schematic presentation of the chromosome 7 directional genomic hybridization (dGH™) assay. **b** In vitro visualization of the inversion in patient III-48 using a directional genomic hybridization (dGH™) assay. The inverted fragment is observed as a signal switch between the sister chromatids (arrowhead in the magnified image **c**). No signal is present in the normal chromosome 7 (magnified image **d**)

We successfully visualized the inv(7) in lymphoblast cells of patient III-48 (Fig. 3) using directional genomic hybridization (dGH™) [66]. The inverted chromosomal fragments inherently possess an opposite 5′ → 3′ orientation, resulting in a jump of fluorescent hybridization signal from one sister chromatid to the other. We also confirmed that this structural variation was absent in two healthy relatives who did not carry the disease haplotype (III-23 and III-39).

### Rare variants in *DPP6* are associated with neurodegenerative dementia

To better understand the genetic contribution of *DPP6* to NBD, we performed massive parallel gene resequencing of *DPP6* coding exons and searched for rare, protein changing variants in 558 EOAD (mean onset age 61.6 ± 6.8 years, range 33–70) and 614 (mean onset age 66.1 ± 9.9 years, age range 20–89) FTD patients. We identified two premature termination codon (PTC) mutations, p.E79Gfs*9 and p.Q23O* in two FTD patients, which were absent from 755 matched controls (mean inclusion age 71.6 ± 9.7 years, age range 34–100) (Table 1 and Table S2). In addition, we identified 22 missense variants and a size variable in-frame Gly-insertion/deletion in exon 1 in AD, FTD patients and controls (Table 1, Table S2 and Table S3). We obtained a significant association (SKAT-O) of rare variants in *DPP6* (minor-allele frequency (MAF) < 0.01) in both AD (*n* = 558, *p* = 0.03, OR = 2.21 95% CI 1.05–4.82) and FTD (*n* = 614, *p* = 0.006, OR = 2.59, 95% CI 1.28–5.49) cohorts.

**Table 1:** Rare variants in *DPP6* in Belgian patient cohorts

Patient	Clinical diagnosis	AAO (years)	AAI (years)	g.DNA	c.DNA	Protein	Protein domain
DR1149	FTD	77	79	g.153584782	c.14C>A	p.A5D	IC Is 3
DR1143	AD	58	67	g.153749963	c.58G>A	p.A20T	IC Is 1
				g.153750014	c.109G>A	p.G37S	IC Is 1
DR1350	FTD-PPA		74	g.153750014	c.109G>A	p.G37S	IC Is 1
DR40*	FTD	44	50	g.153750044	c.140G>T	p.R47L	IC Is 1
DR623	FTD	–	71	g.153750044	c.140G>T	p.R47L	IC Is 1
DR1144	AD	59	61	g.153750065	c.160C>G	p.R54G	IC Is 1
DR807	FTD		65	g.153750065	c.160C>G	p.R54G	IC Is 1
DR1351	AD		56	g.153750065	c.160C>G	p.R54G	IC Is 1
DR1352	AD		55	g.153750065	c.160C>G	p.R54G	IC Is 1
DR1147	FTD	65	65	g.153750086_153750091	c.182_187dup	p.G61_G62dup	IC Is 1
DR1145	FTD-ALS		76	g.153750140_153750141	c.235_236dupG	p.E79Gfs*9	IC Is 1
DR1153	FTD		67	g.154143386	c.331G>A	p.V111I	TM
DR1151	FTD		63	g.154172048	c.383A>G	p.K128R	EC
DR1198*	FTD	52		g.154263996	c.622G>C	p.E208Q	EC
DR1150	PPA	80	81	g.154461074	c.685C>A	p.P229T	EC
DR1154	FTD	–	67	g.154461077	c.688C>T	p.Q230*	EC
DR1155	AD	68	68	g.154519535	c.821G>A	p.R274H	EC
DR1156	AD	53		g.154561208	c.965G>A	p.R322H	EC
DR1289	AD		59	g.154564586	c.1070A>G	p.H357R	EC
**DR414**	PPA	57	59	g.154596653	c.1526C>G	p.P509R	EC
**DR1152**	FTD-ALS	75	75	g.154645528	c.1705G>A	p.D569N	EC
DR1353	FTD		65	g.154645534	c.1711A>C	p.K571Q	EC
DR1354	AD		63	g.154645534	c.1711A>C	p.K571Q	EC
DR114	FTD	67	68	g.154667696	c.1964C>T	p.A655V	EC

The table lists phenotypic and genetic information of rare variants (MAF ≤ 1%) found only in patients. *DPP6* sequencing in 82 patients of the original Dutch population-based EOAD cohort, to which the proband (III-47) of family1270 belonged, identified 1 patient carrying the variant p.R322H, not included in statistical analysis. The specific topological domains in which the mutations are detected are indicated: intracellular domain (IC) of isoform 1 (Is 1, NM_130797.3) and isoform 3 (Is 3 NM_001039350.2); transmembrane (TM) and extracellular domain (EC) common to all *DPP6* isoforms. One variant (p.A5D) was found in the alternative coding exon 1 of isoform 3 in a FTD patient (DR1149). Individuals marked in bold have brain autopsy available (Supplementary material Fig. S3–S4), with a definite diagnosis of FTLD-TDP type D (DR40), definite AD (DR414) and FTLD-ALS type B (DR1152). Individuals marked with an asterisk (*) carry a known pathogenic mutation in *VCP* (p.R159H, DR40) and in *C9orf72* (repeat G_4_C_2_ expansion, DR1198). Abbreviations: *AAO* age at onset, *AAI* age at inclusion, *AD* Alzheimer disease, *ALS* amyotrophic lateral sclerosis, *FTD* frontotemporal dementia, *PPA* primary progressive aphasia. Genomic DNA (g.DNA) is the chromosomal position relative to hg19 build of the reference genome

### Rare variants alter DPP6 and K_v_4.2 expression levels in brain tissue of patients

**Fig. 4:**
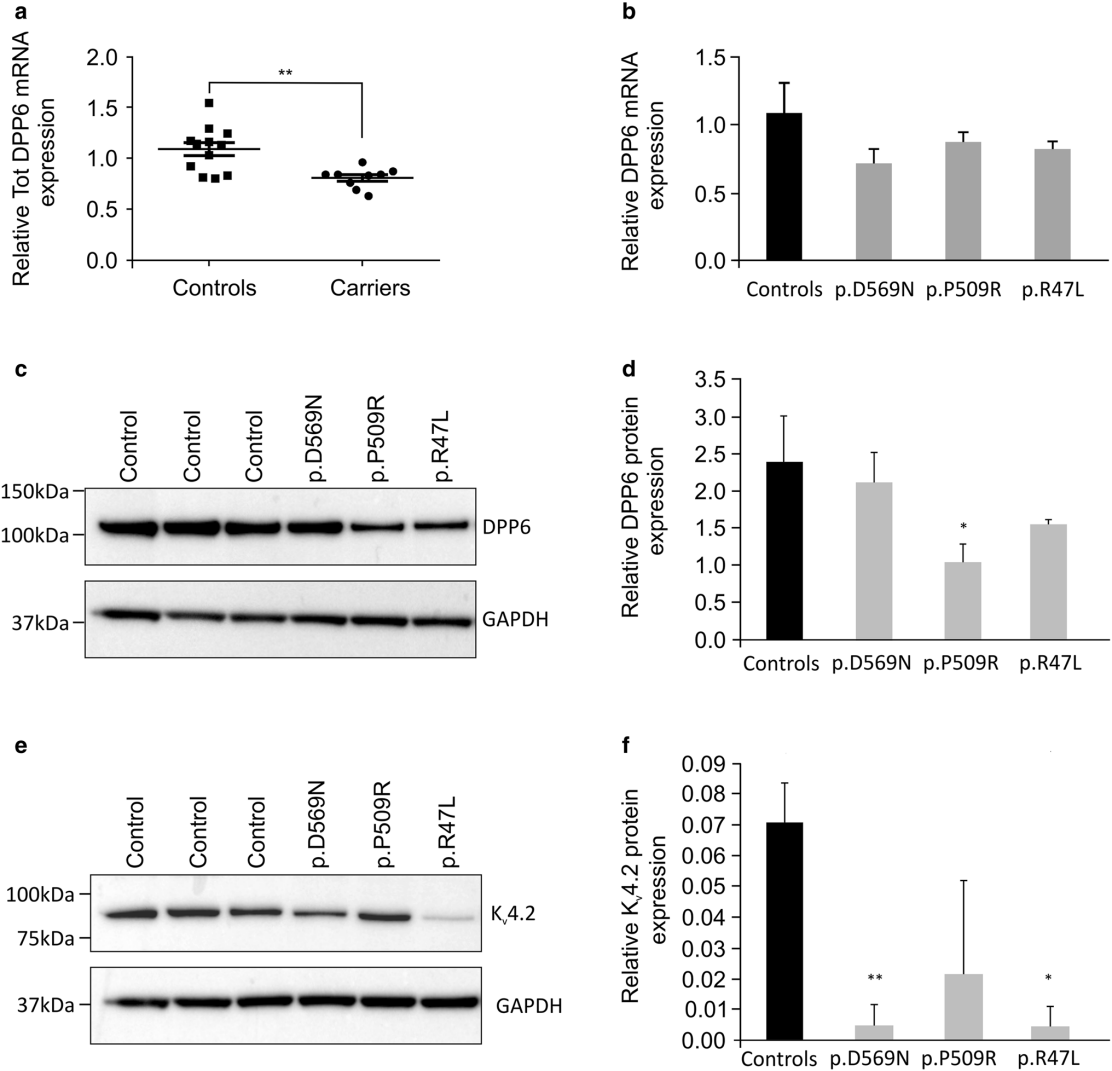
RNA and protein expression levels of DPP6 missense variants. **a** Scatter plot of the *DPP6* mRNA expression levels in patients (*n* = 3) compared to control individuals (*n* = 4). Each circle (patients) or square (control individuals) represents a single measurement; the graph reports the mean ± standard error of the mean (s.e.m.). **p value = 0.0096. **b** DPP6 mRNA expression level results of three experiments for each of the variants (grey bars) compared to averaged data of four controls (black bar). **c** Western blot of *DPP6* carriers and control individuals and **e** western blot of K_v_4.2 in *DPP6* variant carriers and control individuals. **d**, **f** Quantification of the expression levels of DPP6 (**d**) and Kv4.2 (**f**), obtained from pooling two independent protein preparations within the same western blot experiment. Quantifications are shown for each of the missense variants (grey bars) compared to control individuals (*n* = 3, black bar). The relative protein expression is reported as average ± standard deviation of three independent protein preparations and quantifications per sample *p < 0.05, **p < 0.005

Frozen autopsy brain was only available of three patients carrying a *DPP6* missense variant. These patients had a probable clinical diagnosis of primary progressive aphasia (PPA, logopenic variant), FTD (behavior variant) plus Paget’s disease of the bone, and FTD plus ALS (bulbar type) (Table S4).The neuropathological diagnoses were AD (DR414), FTLD-TDP type D (DR40) and FTLD-ALS type B (DR1152) (Fig. S3). Patient DR40 also carried a causal VCP mutation, p.R159H (Table S4). For all three *DPP6* variants p.P509R, p.R47L, and p.D596N, we observed significantly reduced mRNA levels (*p* = 0.0096, Fig. 4a, b), and a marked decrease in DPP6 protein expression levels in the p.P509R and p.R47L carriers (*p* = 0.03) (Fig. 4c, d). Moreover, the protein levels of the potassium channel K_v_4.2, binding partner of DPP6, were also severely reduced (Fig. 4e, f).

### Rare variants in the extracellular domain of DPP6 destabilize the protein and alter its membrane expression

**Fig. 5:**
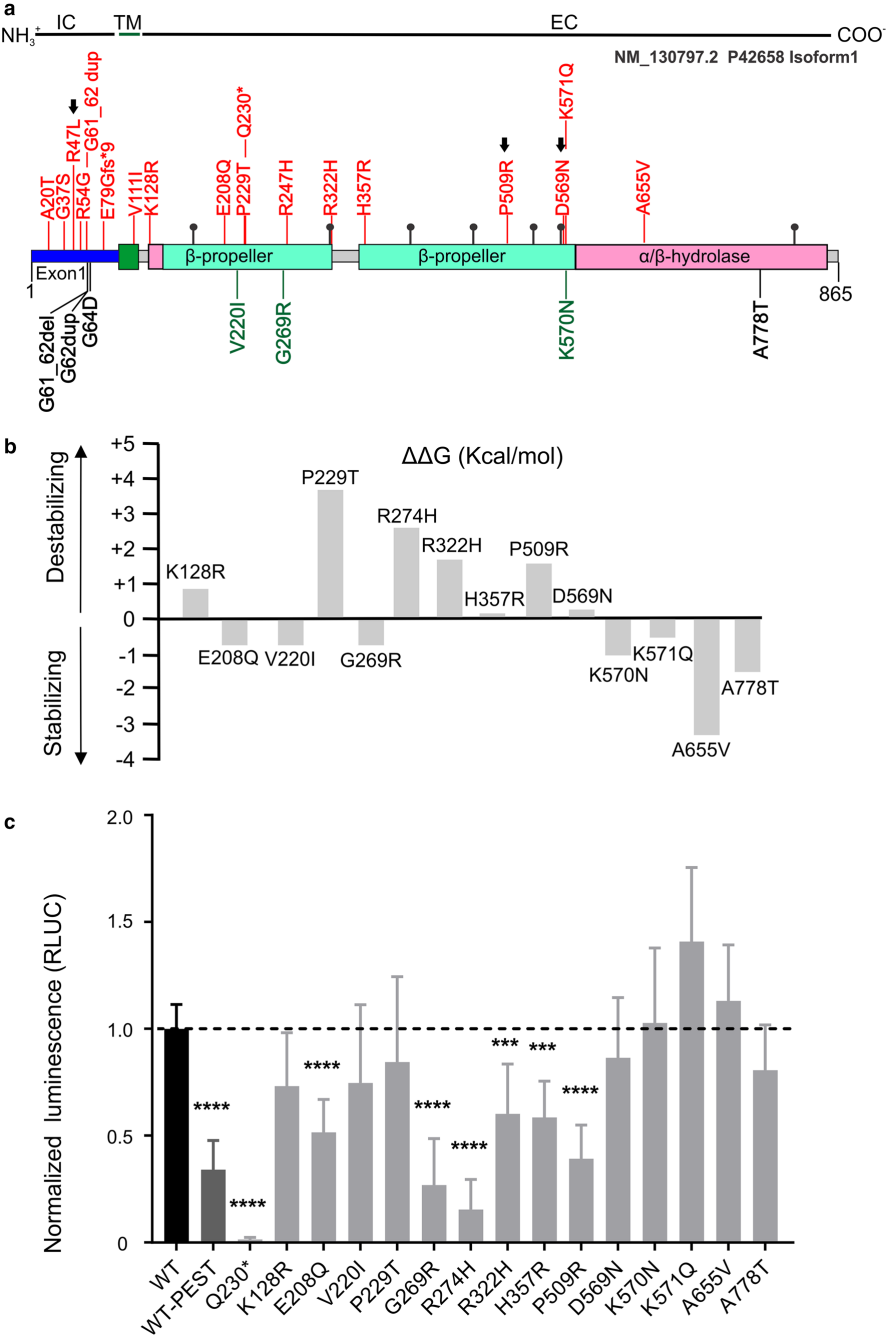
In silico and in vitro modeling of rare variants (MAF < 1%) identified in the screened cohorts. **a** On scale representation of rare variants (MAF < 1%) detected by *DPP6* resequencing. The structural domains [72] are IC, intracellular domain (blue), TM, transmembrane domain (dark green) and EC, extracellular domain including the α/β hydrolase (pink) and the β-propeller domains (turquoise). Seven predicted glycosylation sites are reported as black balls on sticks. DPP6 is a type II transmembrane protein, the N-terminal (NH_3_^+^) and the C-terminal (COO^−^) are marked. Variants located in the transmembrane domain (dark green) and extracellular domain are common to all DPP6 isoforms here are represented on the canonical isoform (NM_130797.2). Variants in the intracellular domain (exon 1) are isoform specific. Apart from variants in the variable intracellular domain in the canonical isoform NM_130797.2 (isoform 1), we detected one additional variant (p.A5D) in exon 1 of isoform 3 (NM_001039350.2) not represented in the figure. Represented in red are variants identified in patients only, in green are depicted the variants identified in control individuals only and in black in both patients and control individuals. Variants marked with a black arrow were included in expression studies, since brain tissue of the carriers was available. **b** Prediction of protein stability in the presence of the missense variants measured in differences in free Gibbs energy (ΔΔ*G*). Destabilizing or stabilizing variants result in positive or negative values, respectively. **c** In vitro protein stability assay using HiBiT-tagged constructs carrying the variants of interest compared to wild-type DPP6. DPP6 fused to the PEST sequence (WT-PEST) was used as positive control. Graph bars represent normalized luminescence (RLUC) that were used to compare the mutated constructs with the wild-type DPP6. Reported data are the pooled results of six independent experiments, error bars represent standard deviation. ***p < 0.001; ****p < 0.0001

In silico modeling of the missense variants located in the extracellular domain of DPP6, predicted a destabilizing effect, measured in positive values of free Gibbs energy, for 7/10 (70%) of the missense variants found in patients only (Table 1, Table S3, Fig. 5). The three variants (p.V220I, p.G269R and K570N), found in controls only, were all predicted to stabilize the protein (ΔΔ*G* < −1). A similar stabilizing effect was detected for the variant p.A778T found in both patients and controls. Furthermore, investigation of the intramolecular interaction with functional residues showed that two missense variants (p.R322H and p.D569N), detected only in patients, could have a deleterious effect on protein glycosylation, either because of the conformational location nearby the canonical glycosylated residue, as p.R322H is in the vicinity of the glycosylation site N319, or because of the amino acid change itself (p.D569N), which could compete for the glycosylation with the closely localized glycosylation site N566 (Fig. S5). Similar to p.R322H, the variant p.K571Q, present only in patients, could compromise the glycosylation of residue N173 (Fig. S6).

Since DPP6 is known to localize on the plasma membrane, we monitored DPP6 stability as changes in DPP6 abundance on the plasma membrane due to folding properties or stability issues or retention in one of the organelles (e.g., endoplasmic reticulum) in the presence of missense variants. To this end, we generated C-terminally Nano-Glo^®^ HiBiT tagged DPP6 constructs and compared wild-type against its missense variants in HEK293T cells. The wild-type construct fused with the PEST sequence, promoting an accelerated degradation, was used as an internal control and the p.Q230* construct was used as a negative control. Of the 14 modeled missense variants located in the extracellular domain of DPP6 (Table 1, Table S2, Fig. 5a), we observed a significant reduced expression (*p* value < 0.001, Fig. 5) on the plasma membrane for 5/10 (50%) of the variants found in patients only (Table 1, Fig. 5). Their CADD scores range from 15.06 for p.E208Q to 34 for p.R247H. The latter variant showed the most drastic reduction of the plasma membrane expression of DPP6 next to the p.Q230*, which was not expressed at all. A significant reduction was observed for one variant (p.G269R) found in controls only. No significant differences were recorded for the variants starting from amino acid position 570. Western blot analysis of the overall DPP6 protein expression showed that the detected differences in DPP6 plasma membrane expression levels were not due to direct changes in total protein abundance (Fig. S7). Moreover, immunofluorescence staining showed proper protein localization on the plasma membrane (Fig. S8).

## Discussion

### The 4 Mb inversion at 7q36 is disrupting *DPP6* causing dementia in family 1270

Our family-based genetic and genomic investigation evidenced the presence of a 4 Mb paracentric inversion at 7q36, segregating on the disease haplotype and explaining the linkage in family 1270. This chromosomal rearrangement was detected by two independent sequencing technologies. Furthermore, the direct long-read sequencing on PromethION, mapped the two breakpoints on chr7:149,704,610–153,786,893. The inversion is likely triggered by the presence of inverted paralogous LCRs (IP-LCRs) with high (> 98%) homology and located 4 Mb apart. This notion is supported both by our DNA local alignments and the data of a genome-wide IP-LCRs search, demonstrating that the *DPP6* locus is enriched for IP-LCRs [17]. IP-LCRs can cause genomic instability by non-allelic homologous recombination (NAHR) mediated by the inversion [17, 22] and this can be associated with disease traits [17, 21]. The family 1270 inversion breakpoint in *DPP6,* is predicted to prevent the transcription of the mutant allele leading to loss of *DPP6*, suggesting that the underlying disease mechanism in family 1270 is haploinsufficiency. The absence in the short- and long-read WGS datasets of the inversion observed in family 1270, is an indication that this inversion is most likely a rare event. The genomic region at 7q36 is known to be vulnerable for structural alterations and chromosomal rearrangements such as copy number variations and translocations [42, 43, 47, 48, 63]. Different breakpoints have been associated with other disease phenotypes including neurodevelopmental disorders [43, 47, 63]. Each of these genomic rearrangements was affecting a single family or a few patients. Larger datasets of long-read WGS and new bioinformatics tools are needed to obtain a more accurate measure of the frequency of structural variants in the 7q36 region [13, 14].

### Genetic association of DPP6 in dementia

To better understand the genetic contribution of *DPP6* to NBD and to support haploinsufficiency as the mode of action, we re-sequenced the coding region of *DPP6* and identified nonsense and frameshift variants, and several missense and short indels that were scattered over the whole *DPP6* gene in patients. The p.Q230* and p.E79Gfs*9 variants, were found in a FTD and FTD-ALS patient who were 67 and 76 years at inclusion in the FTD cohort. These PTC variants likely lead to DPP6 haploinsufficiency through nonsense-mediated decay (NMD) of their mutated transcript. While we did not have brain tissue of the two PTC carriers, an independent study confirmed NMD of a DPP6 PTC variant with 41% reduction of DPP6 transcript levels in the cortex of a definite FTLD patient supporting the loss of DPP6 as the underlying biological mechanism [62].

Since we lacked biosamples to confirm transcript degradation under physiological conditions, mutation modeling of the p.Q230* PTC variant located in the extracellular domain of DPP6, supported the loss of DPP6 protein. In controls, the missense and indel variants were mainly clustering in the non-conserved intracellular protein domain (exon 1) while PTC variants were not observed. The carriers of *DPP6* variants observed only in patients, had an average onset age of 62.9 (*n* = 12, range 44–77) and an inclusion age of 66.1 (*n* = 22, range 50–81) years, which is comparable to the ages and age range observed in family 1270, which is 66.8 ± 7.4 years (*n* = 13; range 47–77). Highly variable onset ages have been reported in patients and families with mutations leading to loss-of-protein, i.e., in *GRN* [10] in FTD and in ATP-binding cassette subfamily a member 7 gene (*ABCA7*) [74] in AD. These genes also show a wide spectrum of mutations having different effects on expression and found over a larger onset age range from early- to late-onset [8, 74]. The *GRN* and *ABCA7* findings indicate that these mutations have different risk contributions to disease that can vary from high to low penetrance [56, 79] which could be valid also for DPP6 missense variants. Since the extracellular domain of DPP6 is highly structured, missense variants in this domain could affect the protein conformation, its function or the interaction with additional proteins. Additional studies are needed to further understand how these variants act. In our study, we did not identify a correlation between *DPP6* rare variants and a specific phenotypic trait including age at onset, disease duration and family history. However, taken the small number of patients in our cohort and the rarity of DPP6 variants, the analysis was likely underpowered and additional studies will be needed. Furthermore, the SKAT-O analysis showed an enrichment of *DPP6* rare variants in both AD and FTD patients, with a stronger significance level in FTD (*p* = 0.006), in which we also identified the PTC variant carriers (0.3%, 2/614). This is in line with an independent genome-wide association study on whole genome sequencing on FTD with TDP-43 (*TAR DNA binding protein 43*) pathology [62]. Two common SNPs in intron 1 of *DPP6* showed genome-wide significant association (rs4726389 *p* = 4.63e−8, OR = 2.45 and rs118113626 *p* = 4.88e−08, OR = 2.48) [62]. Moreover, one PTC carrier was identified amongst the FTLD-TDP43 patients and never in controls, thus matching our findings [62].

### Functional and expression analyses support DPP6 loss as disease mechanism

Understanding the effect of missense variants, compared to PTC mutations, is not trivial. We used the Nano-Glo^®^ HiBiT assay to further characterize the missense variants we identified in *DPP6*. We monitored the changes in DPP6 abundance on the plasma membrane, as in silico analysis predicted differences in protein stability due to alterations in folding properties in 7/10 variants found in patients only in the extracellular domain. The in vitro modeling showed that five variants (p.E208Q, p.R274H, p.R322H, p.H357R and p.509R) identified in patients only, destabilize the protein leading to a reduced level on the plasma membrane, suggesting a loss of function. This was also detected in one variant (p.G269R) found in a control person, suggesting that the missense variants might have a different risk contribution and that the mode of action of missense variants can involve different mechanisms, e.g., the protein function, interaction with other proteins not investigated by this assay. The predictions and the in vitro experiment did not completely overlap, stressing the need for in vitro validation in support of in silico prediction analyses. The in vitro assay correlates with the DPP6 protein levels measured in brain of two carriers of a missense variant. In fact, DR414, carrier of p.P509R, showed reduced DPP6 brain protein expression, in accordance to the protein destabilization detected in vitro. While in the brain tissue of DR1152, carrier of the p.D569N, there were no evident reductions, in agreement with the in vitro assay, suggesting a possible alternative mode of action of this specific variant, for example protein glycosylation. This is supported by the alterations detected in brain expression levels of the K_v_4.2 for this variant carrier. The fact that 50% of the patient-specific variants that we modeled in vitro, have a deleterious effect is a relevant consideration to make, because of the highly structured extracellular domain of DPP6 [72], which is crucial for the protein expression and function, with the different protein domains of DPP6 responsible for its protein localization [44]. In light of this, we suggest a loss of function effect also for these missense variants located in the extracellular domain.

In terms of protein function, DPP6 belongs to the dipeptidyl-peptidase protein family, but lacks protease activity because of a serine into aspartic acid change in the catalytic peptidase domain [72]. By binding, most likely, at the permeation and gating modules [35] of the potassium channel K_v_4.2, DPP6 enhances its expression and regulates its gating properties [35, 51, 52, 73] and it is known to control the dendritic excitability of the hippocampal neurons [73] and the neuronal plasticity [36]. DPP6 and K_v_4.2 directly interact in a multimeric protein complex, in which K_v_4.2 is additionally bound to the auxiliary potassium channel interacting proteins (KChIP) [33]. Reduced potassium and persistent enhanced sodium currents converge to produce neuronal hyperexcitability [81]. A recent study on *Dpp6* knockout (KO) mice suggested a structural function in the formation of filopodia, the precursor of the dendritic spines, and in cellular stability through the binding to the extracellular matrix, thus directly affecting dendritic arborization, spine density and synaptic function [46]. Furthermore, DPP6 loss has been shown to determine memory and learning impairments in young Dpp6-KO mice [45]. Patients affected by anti-DPPX syndrome, with autoantibodies targeting DPP6 and causing reduced protein expression, have memory deficits and neuronal hyperexcitability, features that are improved when DPP6 expression levels are increased [27]. Taken together our data and these studies point toward a deleterious effect of DPP6 loss and support its contribution to dementia.

## Conclusions

Our investigations show that the loss of DPP6 can occur on different levels: the genomic level, with the inversion disrupting the coding sequence; the genetic level with the identification of PTC and deleterious missense variants as well as the protein level, where different variants show a spectrum of alteration in the cellular surface expression. The genetic association with rare variants is an additional line of genetic evidence to link DPP6 to dementia. Our assays to model the missense variants suggest that not all variants identified are deleterious, like the variants located in the ɑ/β hydrolase domain (e.g., p.A778T), This is not unexpected, knowing that widely accepted causal genes for dementia are known to harbor benign variants [9].

The involvement of DPP6 in diverse and independent cellular pathways including neurogenesis and neuronal excitability, could explain why loss of DPP6 was associated with autosomal dominant microcephaly with mental retardation [43] and other neurodevelopmental disorders, including Gilles de la Tourette syndrome (TS) [63] and autism spectrum disorders (ASD) [47]. Heterozygous loss of DPP6 may not represent a single cause of severe intellectual disability but it is likely a susceptibility factor to this phenotype [63]. Currently the link between neurodevelopment and neurodegeneration is unclear, but parallels between the two mechanisms have been proposed [82].

Alterations in the homeostasis of neuronal firing [24] and early neuronal network dysfunctions [58, 84] are emerging concepts in neurodegenerative brain diseases. The results of our genomic, genetic, expression and modeling analyses, provide direct evidence to support the involvement of DPP6 loss in dementia, with loss of function variants (PTC, inversion) having a higher penetrance and disease impact and missense variants having a variable risk contributions to disease from high to low penetrance [56, 79]. Additional studies are needed to fully understand the role of these variants in the disease etiology. Our findings on *DPP6*, as a novel genetic factor in dementia, provide supportive evidence to the emerging concept that neuronal hyperexcitability and alteration of the homeostasis of neuronal firing represent a relevant disease mechanism warranting further investigation.

## Electronic supplementary material

Below is the link to the electronic supplementary material.

Supplementary material 1 (DOCX 13914 kb)

## References

[1] Aronesty E (2013). Comparison of sequencing utility programs. Open Bioinform J.

[2] Auton A, Brooks LD, Durbin RM, Garrison EP, Kang HM, Korbel JO (2015). A global reference for human genetic variation. Nature.

[3] Baker M, Mackenzie IR, Pickering-Brown SM, Gass J, Rademakers R, Lindholm C (2006). Mutations in progranulin cause tau-negative frontotemporal dementia linked to chromosome 17. Nature.

[4] Bettens K, Brouwers N, Van Miegroet H, Gil A, Engelborghs S, De Deyn PP (2010). Follow-up study of susceptibility loci for Alzheimer’s disease and onset age identified by genome-wide association. J Alzheimers Dis.

[5] Busche MA, Konnerth A (2016). Impairments of neural circuit function in Alzheimer’s disease. Philos Trans R Soc Lond B Biol Sci.

[6] Cacace R, Sleegers K, Van Broeckhoven C (2016). Molecular genetics of early-onset Alzheimer disease revisited. Alzheimers Dement.

[7] Cochran JN, Hall AM, Roberson ED (2014). The dendritic hypothesis for Alzheimer’s disease pathophysiology. Brain Res Bull.

[8] Cruts M, Gijselinck I, van der Zee J, Engelborghs S, Wils H, Pirici D (2006). Null mutations in progranulin cause ubiquitin-positive frontotemporal dementia linked to chromosome 17q21. Nature.

[9] Cruts M, Theuns J, Van Broeckhoven C (2012). Locus-specific mutation databases for neurodegenerative brain diseases. Hum Mutat.

[10] Cruts M, Van Broeckhoven C (2008). Loss of progranulin function in frontotemporal lobar degeneration. Trends Genet.

[11] Cruts M, van Duijn CM, Backhovens H, Van den BM, Wehnert A, Serneels S (1998). Estimation of the genetic contribution of presenilin-1 and -2 mutations in a population-based study of presenile Alzheimer disease. Hum Mol Genet.

[12] De Coster W, D’Hert S, Schultz DT, Cruts M, Van Broeckhoven C (2018). NanoPack: visualizing and processing long-read sequencing data. Bioinformatics.

[13] De Coster W, De Roeck A, De Pooter T, D’Hert S, De Rijk P, Strazisar M (2018). Structural variants identified by oxford nanopore PromethION sequencing of the human genome. bioRxiv.

[14] De Roeck A, De Coster W, Bossaerts L, Cacace R, De Pooter T, Van Dongen J (2018). Accurate characterization of expanded tandem repeat length and sequence through whole genome long-read sequencing on PromethION. bioRxiv.

[15] DePristo MA, Banks E, Poplin R, Garimella KV, Maguire JR, Hartl C (2011). A framework for variation discovery and genotyping using next-generation DNA sequencing data. Nat Genet.

[16] Dickerson BC, Salat DH, Greve DN, Chua EF, Rand-Giovannetti E, Rentz DM (2005). Increased hippocampal activation in mild cognitive impairment compared to normal aging and AD. Neurology.

[17] Dittwald P, Gambin T, Gonzaga-Jauregui C, Carvalho CM, Lupski JR, Stankiewicz P (2012). Inverted low-copy repeats and genome instability—a genome-wide analysis. Hum Mutat.

[18] Drmanac R, Sparks AB, Callow MJ, Halpern AL, Burns NL, Kermani BG (2010). Human genome sequencing using unchained base reads on self-assembling DNA nanoarrays. Science.

[19] Engelborghs S, Dermaut B, Goeman J, Saerens J, Marien P, Pickut BA (2003). Prospective Belgian study of neurodegenerative and vascular dementia: APOE genotype effects. J Neurol Neurosurg Psychiatry.

[20] Engelborghs S, Dermaut B, Marien P, Symons A, Vloeberghs E, Maertens K (2006). Dose dependent effect of APOE epsilon4 on behavioral symptoms in frontal lobe dementia. Neurobiol Aging.

[21] Feuk L (2010). Inversion variants in the human genome: role in disease and genome architecture. Genome Med.

[22] Feuk L, Carson AR, Scherer SW (2006). Structural variation in the human genome. Nat Rev Genet.

[23] Folstein MF, Folstein SE, McHugh PR (1975). “Mini-mental state”. A practical method for grading the cognitive state of patients for the clinician. J Psychiatr Res.

[24] Frere S, Slutsky I (2018). Alzheimer’s disease: from firing instability to homeostasis network collapse. Neuron.

[25] Gijselinck I, Van Langenhove T, van der Zee J, Sleegers K, Philtjens S, Kleinberger G (2012). A C9orf72 promoter repeat expansion in a Flanders-Belgian cohort with disorders of the frontotemporal lobar degeneration-amyotrophic lateral sclerosis spectrum: a gene identification study. Lancet Neurol.

[26] Goossens D, Moens LN, Nelis E, Lenaerts AS, Glassee W, Kalbe A (2009). Simultaneous mutation and copy number variation (CNV) detection by multiplex PCR-based GS-FLX sequencing. Hum Mutat.

[27] Hara M, Arino H, Petit-Pedrol M, Sabater L, Titulaer MJ, Martinez-Hernandez E (2017). DPPX antibody-associated encephalitis: main syndrome and antibody effects. Neurology.

[28] Herms J, Dorostkar MM (2016). Dendritic spine pathology in neurodegenerative diseases. Annu Rev Pathol.

[29] Hofman A, Grobbee DE, de Jong PT, van den Ouweland FA (1991). Determinants of disease and disability in the elderly: the Rotterdam Elderly Study. Eur J Epidemiol.

[30] Hofman A, Schulte W, Tanja TA, van Duijn CM, Haaxma R, Lameris AJ (1989). History of dementia and Parkinson’s disease in 1st-degree relatives of patients with Alzheimer’s disease. Neurology.

[31] Honig LS, Mayeux R (2001). Natural history of Alzheimer’s disease. Aging (Milano).

[32] Hyman BT, Phelps CH, Beach TG, Bigio EH, Cairns NJ, Carrillo MC (2012). National institute on aging-Alzheimer’s association guidelines for the neuropathologic assessment of Alzheimer’s disease. Alzheimers Dement.

[33] Jerng HH, Pfaffinger PJ (2014). Modulatory mechanisms and multiple functions of somatodendritic A-type K (+) channel auxiliary subunits. Front Cell Neurosci.

[34] Karch CM, Goate AM (2015). Alzheimer’s disease risk genes and mechanisms of disease pathogenesis. Biol Psychiatry.

[35] Kaulin YA, De Santiago-Castillo JA, Rocha CA, Nadal MS, Rudy B, Covarrubias M (2009). The dipeptidyl-peptidase-like protein DPP6 determines the unitary conductance of neuronal Kv4.2 channels. J Neurosci.

[36] Kim J, Nadal MS, Clemens AM, Baron M, Jung SC, Misumi Y (2008). Kv4 accessory protein DPPX (DPP6) is a critical regulator of membrane excitability in hippocampal CA1 pyramidal neurons. J Neurophysiol.

[37] Kircher M, Witten DM, Jain P, O’Roak BJ, Cooper GM, Shendure J (2014). A general framework for estimating the relative pathogenicity of human genetic variants. Nat Genet.

[38] Kleinberger G, Wils H, Ponsaerts P, Joris G, Timmermans JP, Van Broeckhoven C (2010). Increased caspase activation and decreased TDP-43 solubility in progranulin knockout cortical cultures. J Neurochem.

[39] Krieger E, Vriend G (2014). YASARA view—molecular graphics for all devices—from smartphones to workstations. Bioinformatics.

[40] Lek M, Karczewski KJ, Minikel EV, Samocha KE, Banks E, Fennell T (2016). Analysis of protein-coding genetic variation in 60,706 humans. Nature.

[41] Li H, Durbin R (2009). Fast and accurate short read alignment with Burrows–Wheeler transform. Bioinformatics.

[42] Li L, Chen H, Yin C, Yang C, Wang B, Zheng S (2014). Mapping breakpoints of a familial chromosome insertion (18,7) (q22.1; q36.2q21.11) to DPP6 and CACNA2D1 genes in an azoospermic male. Gene.

[43] Liao C, Fu F, Li R, Yang WQ, Liao HY, Yan JR (2013). Loss-of-function variation in the DPP6 gene is associated with autosomal dominant microcephaly and mental retardation. Eur J Med Genet.

[44] Lin L, Long LK, Hatch MM, Hoffman DA (2014). DPP6 domains responsible for its localization and function. J Biol Chem.

[45] Lin L, Murphy JG, Karlsson RM, Petralia RS, Gutzmann JJ, Abebe D (2018). DPP6 loss impacts hippocampal synaptic development and induces behavioral impairments in recognition, Learning and Memory. Front Cell Neurosci.

[46] Lin L, Sun W, Throesch B, Kung F, Decoster JT, Berner CJ (2013). DPP6 regulation of dendritic morphogenesis impacts hippocampal synaptic development. Nat Commun.

[47] Marshall CR, Noor A, Vincent JB, Lionel AC, Feuk L, Skaug J (2008). Structural variation of chromosomes in autism spectrum disorder. Am J Hum Genet.

[48] Maussion G, Cruceanu C, Rosenfeld JA, Bell SC, Jollant F, Szatkiewicz J (2017). Implication of LRRC4C and DPP6 in neurodevelopmental disorders. Am J Med Genet A.

[49] McKhann G, Drachman D, Folstein M, Katzman R, Price D, Stadlan EM (1984). Clinical diagnosis of Alzheimer’s disease: report of the NINCDS-ADRDA work group under the auspices of department of health and human services task force on Alzheimer’s disease. Neurology.

[50] McKhann GM, Knopman DS, Chertkow H, Hyman BT, Jack CR, Kawas CH (2011). The diagnosis of dementia due to Alzheimer’s disease: recommendations from the National Institute on Aging-Alzheimer’s Association workgroups on diagnostic guidelines for Alzheimer’s disease. Alzheimers Dement.

[51] Nadal MS, Amarillo Y, Vega-Saenz de Miera E, Rudy B (2001). Evidence for the presence of a novel Kv4-mediated A-type K(+) channel-modifying factor. J Physiol.

[52] Nadal MS, Ozaita A, Amarillo Y, Vega-Saenz de Miera E, Ma Y, Mo W (2003). The CD26-related dipeptidyl aminopeptidase-like protein DPPX is a critical component of neuronal A-type K + channels. Neuron.

[53] Nasreddine ZS, Phillips NA, Bedirian V, Charbonneau S, Whitehead V, Collin I (2005). The montreal cognitive assessment, MoCA: a brief screening tool for mild cognitive impairment. J Am Geriatr Soc.

[54] Neary D, Snowden JS, Gustafson L, Passant U, Stuss D, Black S (1998). Frontotemporal lobar degeneration: a consensus on clinical diagnostic criteria. Neurology.

[55] Nestor MW, Hoffman DA (2012). Aberrant dendritic excitability: a common pathophysiology in CNS disorders affecting memory?. Mol Neurobiol.

[56] Nicolas G, Charbonnier C, Campion D (2016). From common to rare variants: the genetic component of Alzheimer disease. Hum Hered.

[57] Noe L, Kucherov G (2005). YASS: enhancing the sensitivity of DNA similarity search. Nucleic Acids Res.

[58] Palop JJ, Mucke L (2016). Network abnormalities and interneuron dysfunction in Alzheimer disease. Nat Rev Neurosci.

[59] Pedersen BS, Quinlan AR (2018). Mosdepth: quick coverage calculation for genomes and exomes. Bioinformatics.

[60] Pongs O, Schwarz JR (2010). Ancillary subunits associated with voltage-dependent K + channels. Physiol Rev.

[61] Pottier C, Ravenscroft TA, Sanchez-Contreras M, Rademakers R (2016). Genetics of FTLD: overview and what else we can expect from genetic studies. J Neurochem.

[62] Pottier C, Ren Y, Perkerson RB, Baker M, Jenkins GD, van Blitterswijk M (2019). Genome-wide analyses as part of the international FTLD-TDP whole-genome sequencing consortium reveals novel disease risk factors and increases support for immune dysfunction in FTLD. Acta Neuropathol.

[63] Prontera P, Napolioni V, Ottaviani V, Rogaia D, Fusco C, Augello B (2014). DPP6 gene disruption in a family with Gilles de la Tourette syndrome. Neurogenetics.

[64] Quiroz YT, Budson AE, Celone K, Ruiz A, Newmark R, Castrillon G (2010). Hippocampal hyperactivation in presymptomatic familial Alzheimer’s disease. Ann Neurol.

[65] Rademakers R, Cruts M, Sleegers K, Dermaut B, Theuns J, Aulchenko Y (2005). Linkage and association studies identify a novel locus for Alzheimer disease at 7q36 in a Dutch population-based sample. Am J Hum Genet.

[66] Rascovsky K, Hodges JR, Knopman D, Mendez MF, Kramer JH, Neuhaus J (2011). Sensitivity of revised diagnostic criteria for the behavioural variant of frontotemporal dementia. Brain.

[67] Ray FA, Zimmerman E, Robinson B, Cornforth MN, Bedford JS, Goodwin EH (2013). Directional genomic hybridization for chromosomal inversion discovery and detection. Chromosome Res.

[68] Reumers J, De Rijk P, Zhao H, Liekens A, Smeets D, Cleary J (2012). Optimized filtering reduces the error rate in detecting genomic variants by short-read sequencing. Nat Biotechnol.

[69] Rosenbloom KR, Sloan CA, Malladi VS, Dreszer TR, Learned K, Kirkup VM (2013). ENCODE data in the UCSC genome browser: year 5 update. Nucleic Acids Res.

[70] Sedlazeck FJ, Rescheneder P, Smolka M, Fang H, Nattestad M, von Haeseler A (2018). Accurate detection of complex structural variations using single-molecule sequencing. Nat Methods.

[71] Shao H, Ganesamoorthy D, Duarte T, Cao MD, Hoggart CJ, Coin LJM (2018). npInv: accurate detection and genotyping of inversions using long read sub-alignment. BMC Bioinform.

[72] Strop P, Bankovich AJ, Hansen KC, Garcia KC, Brunger AT (2004). Structure of a human A-type potassium channel interacting protein DPPX, a member of the dipeptidyl aminopeptidase family. J Mol Biol.

[73] Sun W, Maffie JK, Lin L, Petralia RS, Rudy B, Hoffman DA (2011). DPP6 establishes the A-type K(+) current gradient critical for the regulation of dendritic excitability in CA1 hippocampal neurons. Neuron.

[74] Van den Bossche T, Sleegers K, Cuyvers E, Engelborghs S, Sieben A, De Roeck A (2016). Phenotypic characteristics of Alzheimer patients carrying an ABCA7 mutation. Neurology.

[75] van Duijn CM, Hendriks L, Cruts M, Hardy JA, Hofman A, Van Broeckhoven C (1991). Amyloid precursor protein gene mutation in early-onset Alzheimer’s disease. Lancet.

[76] van Duijn CM, Hendriks L, Farrer LA, Backhovens H, Cruts M, Wehnert A (1994). A population-based study of familial Alzheimer disease: linkage to chromosomes 14, 19, and 21. Am J Hum Genet.

[77] Van Durme J, Delgado J, Stricher F, Serrano L, Schymkowitz J, Rousseau F (2011). A graphical interface for the FoldX forcefield. Bioinformatics.

[78] Van Langenhove T, van der Zee J, Gijselinck I, Engelborghs S, Vandenberghe R, Vandenbulcke M (2013). Distinct clinical characteristics of C9orf72 expansion carriers compared with GRN, MAPT, and nonmutation carriers in a Flanders-Belgian FTLD cohort. JAMA Neurol.

[79] Van Mossevelde S, van der Zee J, Gijselinck I, Engelborghs S, Sieben A, Van Langenhove T (2016). Clinical features of TBK1 carriers compared with C9orf72, GRN and non-mutation carriers in a Belgian cohort. Brain.

[80] Vandesompele J, De Preter K, Pattyn F, Poppe B, Van Roy N, De Paepe A (2002). Accurate normalization of real-time quantitative RT-PCR data by geometric averaging of multiple internal control genes. Genome Biol.

[81] Wainger BJ, Kiskinis E, Mellin C, Wiskow O, Han SS, Sandoe J (2014). Intrinsic membrane hyperexcitability of amyotrophic lateral sclerosis patient-derived motor neurons. Cell Rep.

[82] Zhu GC, Webber KM, Atwood CS, Bowen RL, Perry G, Smith MA, Lajtha A, Perez-Polo JR, Rossner S (2008). Parallels between neurodevelopment and neurodegeneration: a case study of Alzheimer’s disease. Handbook of Neurochemistry and molecular neurobiology.

[83] Zhou J, Greicius MD, Gennatas ED, Growdon ME, Jang JY, Rabinovici GD (2010). Divergent network connectivity changes in behavioural variant frontotemporal dementia and Alzheimer’s disease. Brain.

[84] Zott B, Busche MA, Sperling RA, Konnerth A (2018). What happens with the circuit in Alzheimer’s disease in mice and humans?. Annu Rev Neurosci.

