# Supplementary material for: Loss of DPP6 in neurodegenerative dementia: a genetic player in the dysfunction of neuronal excitability
Source: Acta Neuropathol. 2019 Mar 14;137(6):901–18. doi: 10.1007/s00401-019-01976-3 (PMC6531610; doi:10.1007/s00401-019-01976-3)
Supplement: Supplementary file 1 — Supplementary material 1 (DOCX 13914 kb) [file 401_2019_1976_MOESM1_ESM.docx]

**Loss of *DPP6* in neurodegenerative dementia: a genetic player in the dysfunction of neuronal excitability.**

Rita Cacace^1,2,3^, Bavo Heeman^1,2,3^, Sara Van Mossevelde^1,2,3,4,5^, Arne De Roeck^1,2,3^, Julie Hoogmartens^1,2,3^, Peter De Rijk^1,3^, Helena Gossye^1,2,3,4,5^, Kristof De Vos^1,2,3^, Wouter De Coster^1,3^, Mojca Strazisar^1,3^, Greet De Baets^6,7^, Joost Schymkowitz^6,7^, Frederic Rousseau^6.7^, Nathalie Geerts^1,2,3^, Tim De Pooter^1,3^, Karin Peeters^1,2,3^, Anne Sieben^1,2,8^, Jean-Jacques Martin^2^, Sebastiaan Engelborghs^2,3,5^, Eric Salmon^9^, Patrick Santens^8^, Rik Vandenberghe^10,11^, Patrick Cras^2,3,4^, Peter P De Deyn^2,3,5^, John C. van Swieten^12^, Cornelia M. van Duijn^13^, Julie van der Zee^1,2,3^, Kristel Sleegers^1,2,3^ and Christine Van Broeckhoven^1,2,3#^ on behalf of the BELNEU Consortium.

^1^Center for Molecular Neurology, VIB, Antwerp, Belgium

^2^Institute Born-Bunge, Antwerp, Belgium

^3^University of Antwerp, Antwerp, Belgium

^4^Department of Neurology, Antwerp University Hospital, Edegem, Belgium

^5^Department of Neurology and Memory Clinic, Hospital Network Antwerp (ZNA), Middelheim and Hoge Beuken, Antwerp, Belgium

^6^Switch Laboratory, VIB-KU Leuven Centre for Brain & Disease Research, Leuven, Belgium

^7^Switch Laboratory, Department of Cellular and Molecular Medicine, KU Leuven, Leuven, Belgium

^8^Department of Neurology, University Hospital Ghent and University of Ghent, Ghent, Belgium

^9^Department of Neurology, Centre Hospitalier Universitaire de Liège and University of Liège, Liège, Belgium

^10^Department of Neurosciences, Faculty of Medicine, KU Leuven, Leuven, Belgium

^11^Laboratory of Cognitive Neurology, Department of Neurology, University Hospitals Leuven, Leuven, Belgium

^12^Department of Neurology, Erasmus University Medical Centre, Rotterdam, The Netherlands

^13^Department of Epidemiology, Erasmus University Medical Centre, Rotterdam, The Netherlands

**Belgian Neurology (BELNEU) Consortium side author list:** The following members of the BELNEU consortium have contributed to the clinical and pathological phenotyping and follow-up of the Belgian patients and families: Johan Goeman, Roeland Crols, Dirk Nuytten (Hospital Network Antwerp (ZNA), Antwerp); Jan L. De Bleecker, Tim Van Langenhove (University Hospital Ghent, Ghent); Adrian Ivanoiu (Saint-Luc University Hospital, Brussels); Olivier Deryck, Bruno Bergmans (General Hospital Sint-Jan Brugge, Bruges); Jan Versijpt, Alex Michotte (VUB University Hospital Brussels, Brussels); Jean Delbeck (General Hospital Sint-Maria, Halle), Christiana Willems, Nina De Klippel (General Hospital Jessa Hospital, Hasselt).

**#Corresponding author:**

Prof. Dr. Christine Van Broeckhoven PhD DSc

Neurodegenerative Brain Diseases group, VIB Center for Molecular Neurology

University of Antwerp – CDE, Universiteitsplein 1, B-2610 Antwerp, Belgium

Tel. +32 3 265 1101; Fax. +32 3 265 8410

[christine.vanbroeckhoven@uantwerpen.vib.be](mailto:christine.vanbroeckhoven@uantwerpen.vib.be)

**Fig. S1: Family 1270 extended pedigree**


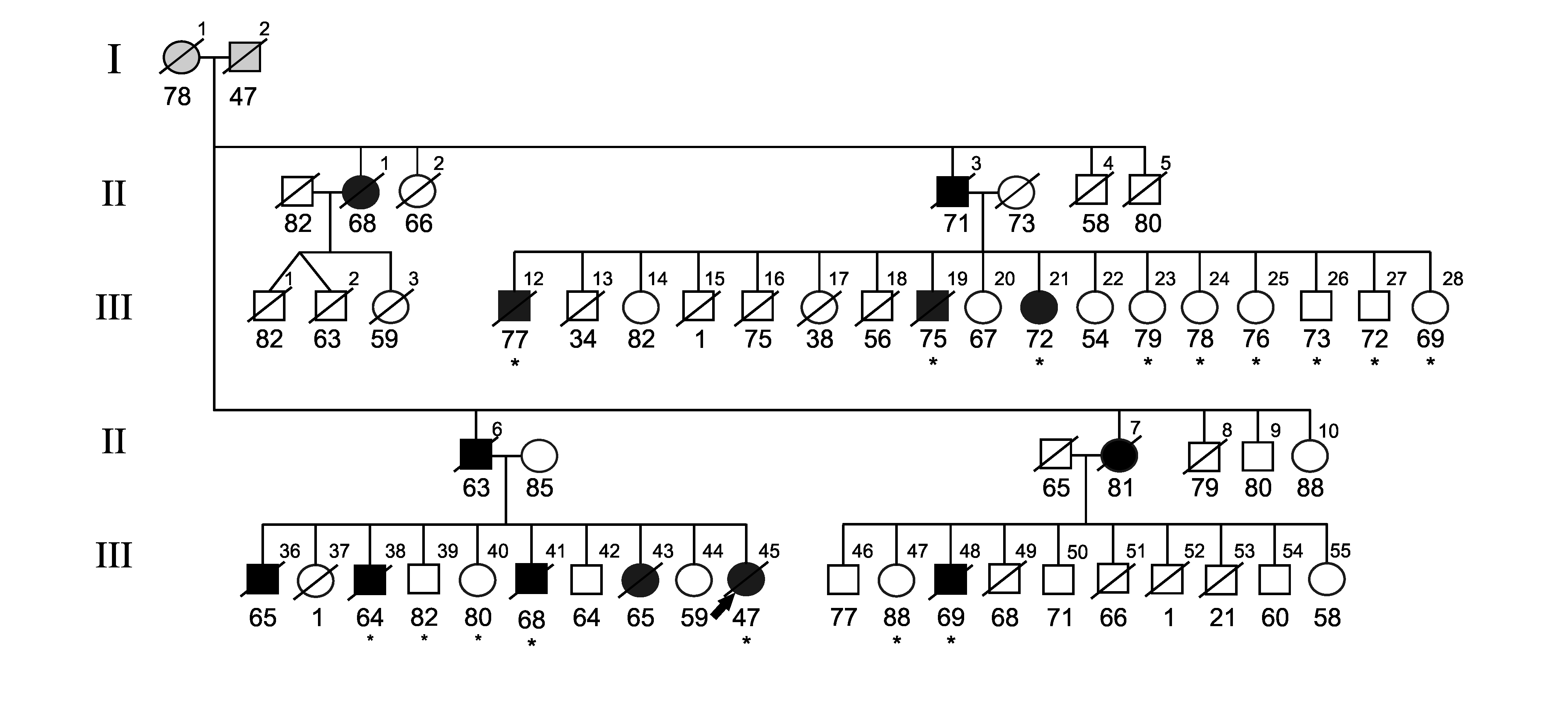


**Fig. S1 legend:** Black symbols represent patients, white symbols represent unaffected or at-risk individuals with unknown phenotype. Arabic numbers above the symbols denote individuals, Arabic numbers below the symbols denote age at onset for patients or either age at last examination or age at death for unaffected individuals and at-risk individuals with unknown phenotype. An asterisk (*) indicates an individual included in the linkage analysis in the previous study. The arrow identifies the proband in the family. Paired end WGS data was generated for patients III-12, III-38, III-41 and III-48. Nanopore PromethION WGS was performed for patient III-48.

Family 1270 was sampled in the 80-90 at a time that the amyloid/tau CSF biomarkers and amyloid imaging were not available hampering clinical diagnosis and as well as subtype differentiation (e.g. [2,5]). In the proband, and most other patients, the disease initially presented with memory impairment, except for one patient, in whom a change of character was the initial complaint, later followed by memory loss. In all patients, the disease progressed into other areas of cognition, such as praxis and speech. Neuroimaging (CT scan) was available only for two patients, age 74 and 82 years, showing cortical atrophy in both patients. The patient who received the diagnosis of possible AD (III-21), had a CT scan at age 82 showing marked cortical and moderate subcortical atrophy, most pronounced in the temporal and frontal regions, with secondary dilatation of the lateral and third ventricles also, mainly frontally localized, periventricular leukoencephalomalacia was detected.

**Brain CT scan of patient III-21 at age 82 years (as published in Rademakers et al., 2005).**


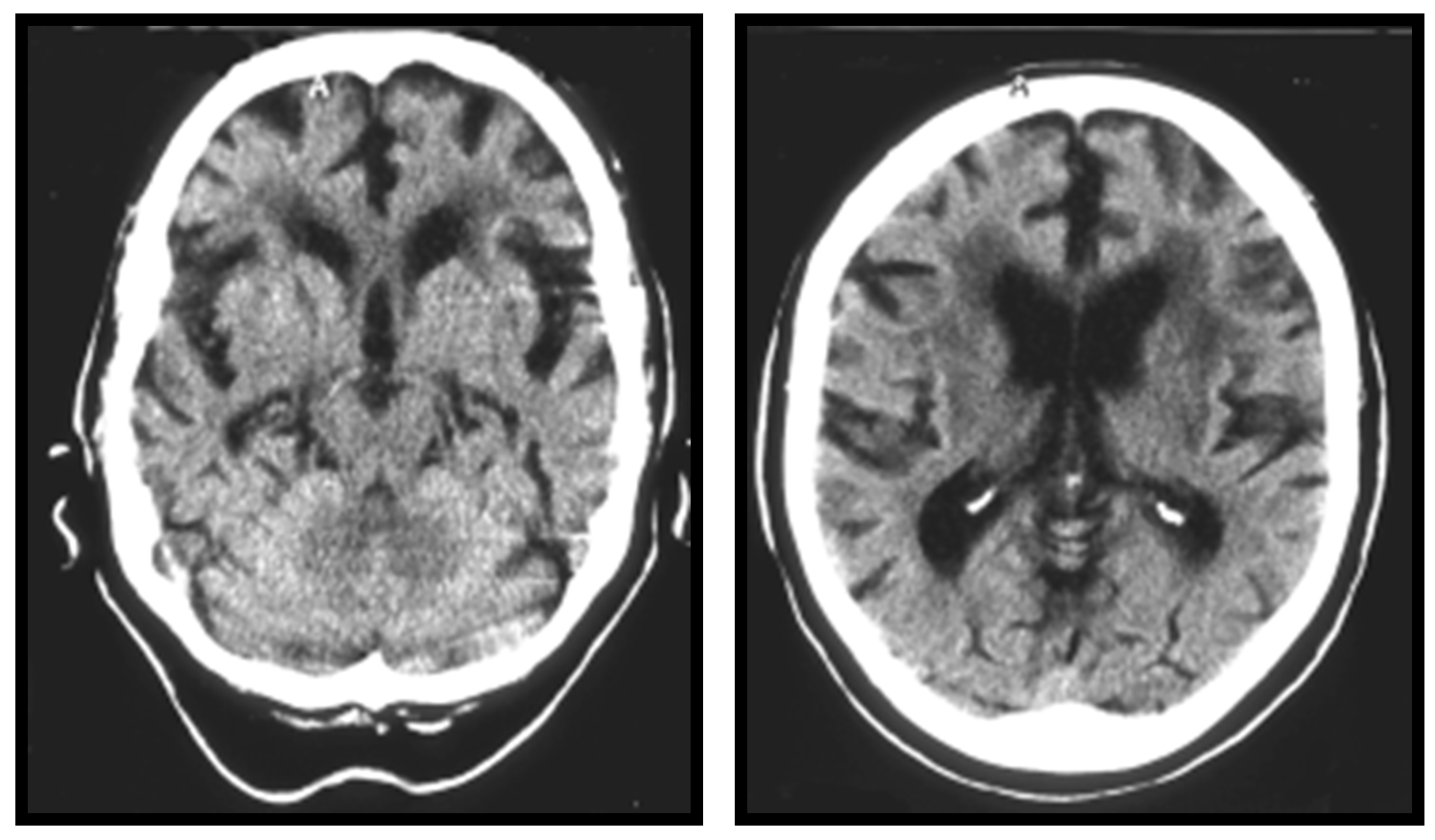


Still today, the differential clinical diagnosis between AD and FTD patients are not evident and need additional biomarkers as well as genetic testing. A comparable story happened for the Dutch family 1083 [6]. Proband 1083 was included in the same Rotterdam genetic epidemiological study of early-onset AD in the Netherlands as family 1270 [4], and was one of the 10 familial probands of whom the relatives were sampled to perform genetic linkage studies [8]. In this autosomal dominant family 1083, mean onset age was 64.9 years, age range 53-79 years. After a clinical follow-up of family 1083, a significant linkage was obtained on chromosome 17q21 (multi-point LOD score of 5.51, candidate region 4.8 cM) [6]. The clinical and neuropathological follow-up of this family showed that the phenotype most closely resembled frontotemporal dementia (FTD). Here, one autopsy brain showed dense ubiquitin-positive neuronal inclusions that were tau negative [6]. Further gene searches in family 1083 in the candidate region at 17q21 identified a nonsense mutation in the *PGRN* gene [3].

| Chromosome | Reference genome hg18 (g.) | Gene Symbol | Protein Change |
| --- | --- | --- | --- |
| 1 | 11777059A>C | *MTHFR* | p.S430R |
| 1 | 11822838C>T | *CLCN6* | p.R861W |
| 1 | 37969757C>T | *EPHA10* | p.V526I |
| 1 | 38256025T>G | *UTP11L* | p.L75R |
| 1 | 246375612C>A | *OR2M5* | p.D180E |
| 1 | 246579307G>C | *OR14C36* | p.G203A |
| 4 | 35962410A>C | *DTHD1* | p.E105A |
| 4 | 38451519C>A | *TLR10* | p.M696I |
| 4 | 52640677G>A | *SPATA18* | p.R397Q |

**Table S1. Family 1270: Coding variants identified genome-wide by WGS with no co-segregation**

**Fig. S2: Inversion detection in paired end short read sequencing in family 1270**


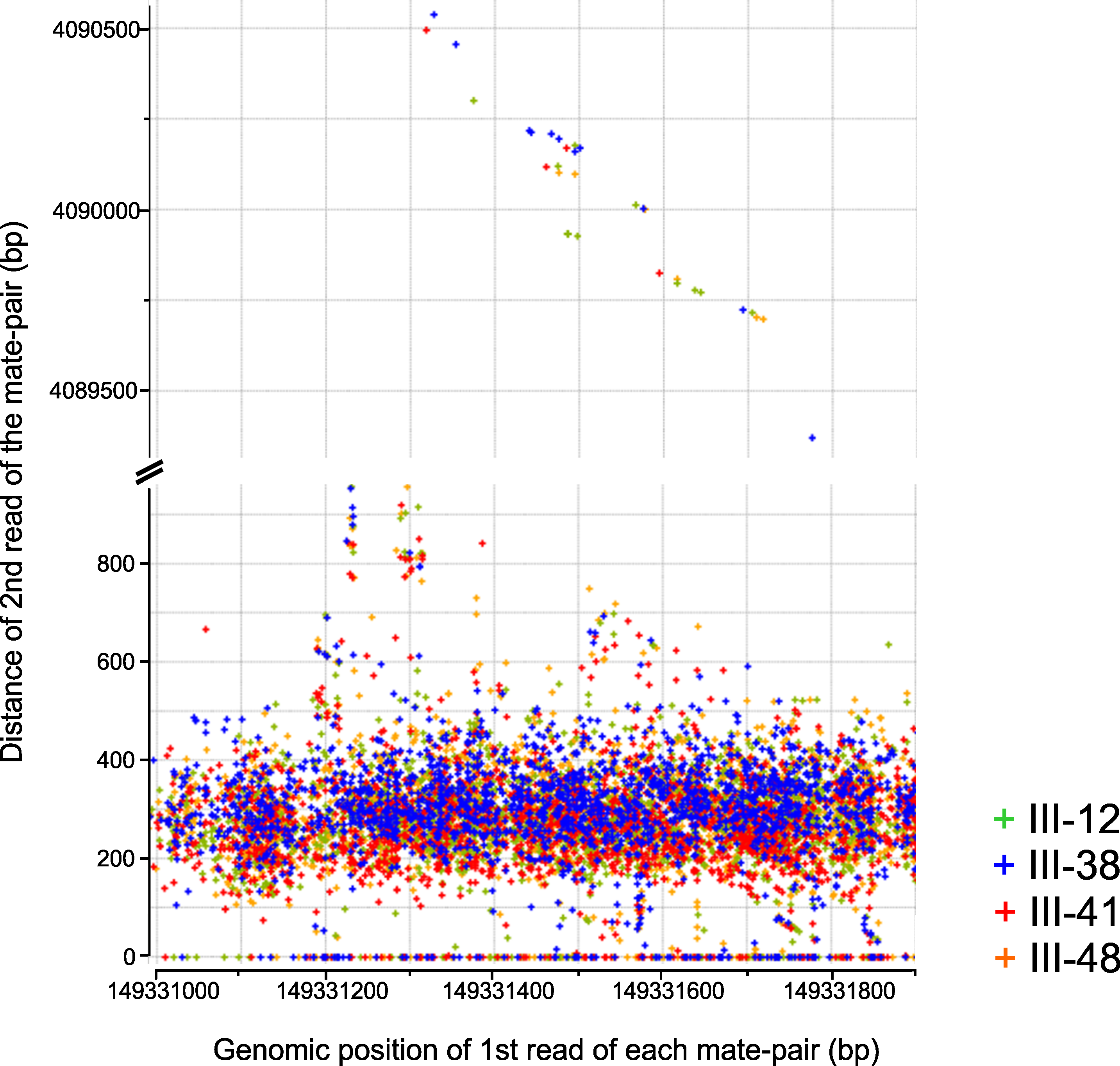


**Fig. S2 legend:** Read pair distribution obtained in WGS of 4 patients (III-12; green, III-38, blue; III-41, red and III-48, orange) of family 1270 (*in silico*). Each cross represents a mate-pair. The X-axis reports the genomic position on chromosome 7 of the first read of the mate-pair while the Y-axis represents the distance to the mapping position of the second read. In the top of the graph the mate pairs are indicative of a *ca.* 4 Mb inversion.

**Table S2**: **Rare variants (MAF<1%) identified in both patients and controls and in controls only.** Abbreviations: AAO: age at onset, AAI: age at inclusion, AD: Alzheimer disease, FTD: frontotemporal dementia, PPA: primary progressive aphasia. Individual DR698.2 marked with an asterisk (*), is the monogenic twin sibling of DR698 and was not included in the statistical analysis. Patient DR1163 marked with an ash (#) has a definite AD pathology. The protein domains are reported, IC= intracellular, EC=extracellular.

| **Protein Domain** | **gDNA (hg19)** | **cDNA** | **Protein** | **CADD (PHRED) score** | **Effect on stability (ΔΔG)** | **dbSNP id** | **ExAc overall (%)** |
| --- | --- | --- | --- | --- | --- | --- | --- |
| Intracellular | g.153584782 | NM_001039350 c.14C>A | p.A5D | 24.8 | NA | **-** | - |
|  | g.153749963 | NM_130797.2 c.58G>A | p.A20T | 12.98 | NA | rs2533731 | - |
|  | g.153750014 | NM_130797.2 c.109G>A | p.G37S | 9.12 | NA | - | - |
|  | g.153750045 | NM_130797.2 c.140G>T | p.R47L | 11.73 | NA | - | - |
|  | g.153750065 | NM_130797.2 c.160C>G | p.R54G | 11.12 | NA | - | - |
|  | g.153750086_153750091 | NM_130797.2: c.182_187del | p.G61_G62del | 11.95 | NA | - | - |
|  | g.153750086_153750091 | NM_130797.2: c.182_187dup | p.G61_G62dup | 8.44 | NA | - | - |
|  | g.153750089_153750091 | NM_130797.2: c.185_187dup | p.G62dup | 8.76 | NA | - | - |
|  | g.153750096 | NM_130797.2 c.191G>A | p.G64D | 21.3 | NA | rs2533730 | 0.375 |
|  | g.153750140_153750141 | NM_130797.2: c.235_236dupG | p.E79Gfs*9 | 27.5 | NA | - | - |
| Transmembrane | g.154143386 | NM_130797.2 c.331G>A | p.V111I | 13.42 | NA | rs370015674 | 0.00006628 |
| Extracellular | g.154172048 | NM_130797.2 c.383A>G | p.K128R | 23.7 | 0.72 | - | - |
|  | g.154263996 | NM_130797 c.622G>C | p.E208Q | 15.06 | -0.64 | - | - |
|  | g.154429561 | NM_130797.2 c.658G>A | p.V220I | 18.71 | -0.6 | - | - |
|  | g.154461074 | NM_130797.2 c.685C>A | p.P229T | 16.47 | 3.7 | - | - |
|  | g.154461077 | NM_130797.2 c.688C>T | p.Q230* | 42 | NA | - | - |
|  | g.154519519 | NM_130797.2 c.805G>A | p.G269R | 23 | -0.6 | - |  |
|  | g.154519535 | NM_130797.2 c.821G>A | p.R274H | 34 | 2.5 | - | 0.00008308 |
|  | g.154561208 | NM_130797.2 c.965G>A | p.R322H | 26.5 | 1.7 | rs368682396 | 0.00 |
|  | g.154564586 | NM_130797.2 c.1070A>G | p.H357R | 18.19 | 0.04 | - | - |
|  | g.154596653 | NM_130797.2 c.1526C>G | p.P509R | 25.2 | 1.6 | - | - |
|  | g.154645528 | NM_130797.2 c.1705G>A | p.D569N | 15.18 | 0.2 | - | 0.00004141 |
|  | g.154645533 | NM_130797.2 c.1710G>T | p.K570N | 10.54 | -1 | - | - |
|  | g.154645534 | NM_130797 c.1711A>C | p.K571Q | 5.42 | -0.5 | rs140460765 | 0.001 |
|  | g.154667696 | NM_130797.2 c.1964C>T | p.A655V | 14.43 | -3.3 | - | 0.0000781 |
|  | g.154681009 | NM_130797.2 c.2332G>A | p.A778T | 15.19 | -1.4 | rs188276022 | 0.001543 |

**Table S3: Prediction of deleteriousness of the nucleotide changes for the *DPP6* variants.** Combined Annotation Dependent Depletion (CADD) v1.3 was used to annotate the DPP6 variants. The rescaled (PHRED) score is reported, which correlates with allelic diversity and variant pathogenicity. ΔΔG: difference in free Gibbs energy, effect on stability is computed using FoldX, positive values indicate destabilization of the protein structure; NA: not available, DPP6 crystallographic structure is available for the extracellular domain only. The dbSNP identifier if available is reported. Minor allele frequency (MAF) in the overall Exome Aggregation Consortium (ExAc) cohort is reported.

| Patient | Clinical | AAO | AAD | Gender | Imaging biomarkers | Family history | Autopsy | Neuropath |
| --- | --- | --- | --- | --- | --- | --- | --- | --- |
| DR414 | Probable PPA type lvPPA | 59 | 66 | Male | - perfusion SPECT: bilateral parietal and occipital, slight temporal hypoperfusion - CT: bilateral temporal-parietal and occipital atrophy | Unknown | IB5941 | AD  A3B3C3 |
| DR40 | Probable bvFTD + Paget’s disease of the bone | 44 | 57 | Male | - CT: periventricular lacunar ischemic lesion, right frontal lobe - MRI: cortico-subcortical atrophy, signs of chronic vascular lesions - perfusion SPECT: relative hypoperfusion frontotemporal lobes, left worse than right; parietal lobes, left worse than right; cerebellum, mostly right. | Proband of VCP p.R159H family [7] . | IB5850 | FTLD TDP  type D |
| DR1152 | Probable FTD +  ALS type bulbar | 75 | 76 | Female | - FDG PET: frontotemporal hypometabolism - MRI: global atrophy | Negative | IB6015 | FTLD TDP  type B  AD  A2B1C2 |

**Table S4: Clinical and neuropathological data of autopsied DPP6 missense mutation carriers**

Abbreviations: AAO = age at onset; AAD; age at death; Neuropath. = neuropathology; PPA = primary progressive aphasia (lvPPA); lvPPA = logopenic variant PPA; bvFTD = behavioral variant Frontotemporal dementia, ALS = amyotrophic lateral sclerosis; SPECT = Single-photon emission computed tomography, CT = computed tomography; MRI = Magnetic resonance imaging; FDG PET = Fludeoxyglucose positron emission tomography.

**Fig. S3. Macroscopic brain examination of missense variants carriers.**


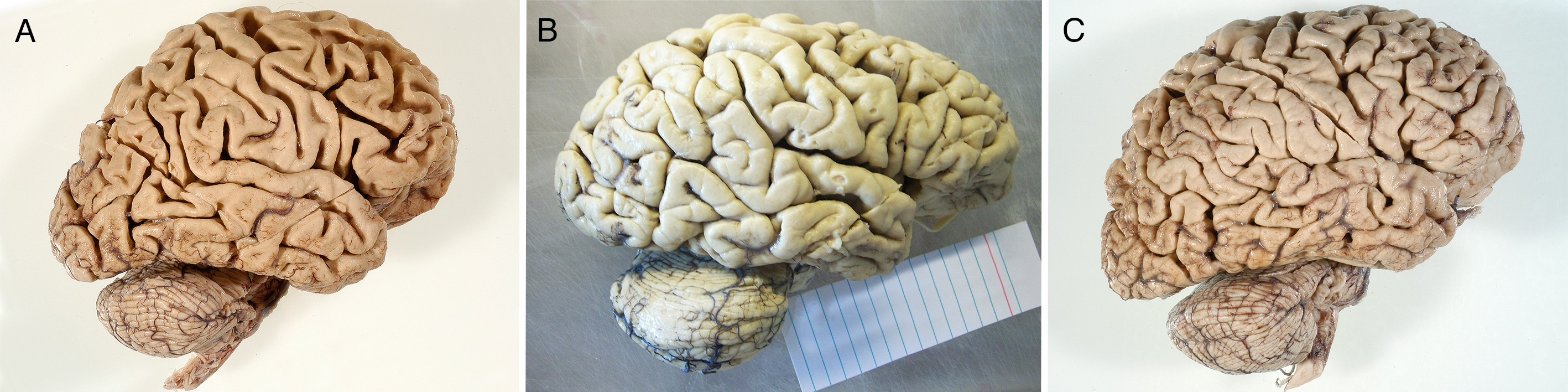


**Fig. S3 legend:** Lateral view of the left hemisphere. A) DR40, generalized cortical atrophy, most pronounced of frontal and temporal cortex. B) DR1152 frontal atrophy is pronounced, with relative sparing of the rest of the cortex. C) DR414 frontoparietal cortical atrophy.

**Fig. S4. Microscopic histological evaluation of DPP6 missense variants carriers.**


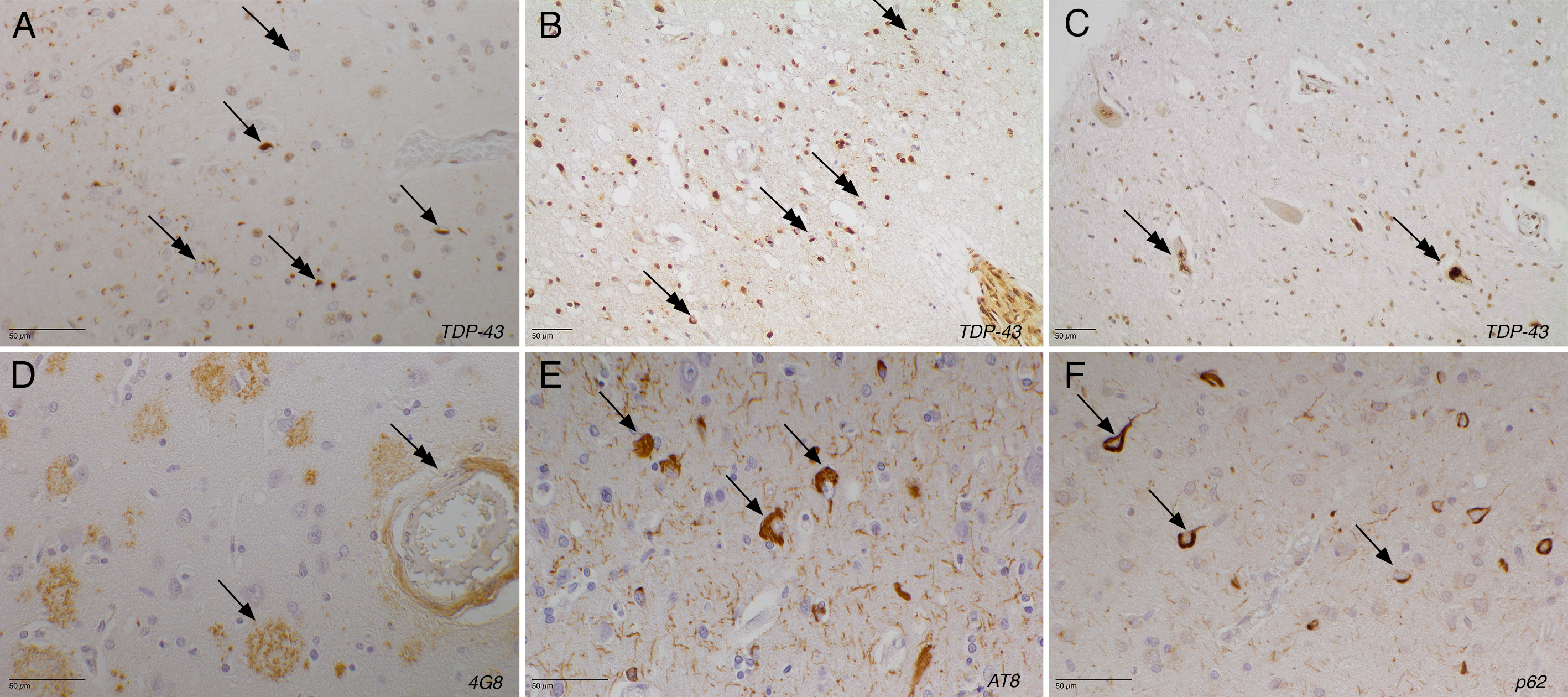


**Fig. S4 legend. Microscopic histological evaluation of DPP6 missense mutation carriers.** A) TDP stain of the superior temporal gyrus of DR40. TDP-43 proteinopathy type D. Arrows show the intranuclear neuronal inclusions (NII), whereas the couble arrows show the intracytoplasmic neuronal inclusions (NCI). B) TDP stain of the frontal cortex area 6 of DR1152. A moderate amount of NCI (double arrow), which have often a granular aspect is present. C) TDP stain of the cervical ventral horn neurons of DR1152. The double arrow indicates the large NCI in the motor neurons. D) 4G8 stain of the frontal cortex area 6 of DR414. There are many classic senile plaques containing beta-amyloid (arrow), as well as beta-amyloid deposits in the arteriolar wall (double arrow). E) AT8 stain of the frontal cortex area 6 of DR414. Arrows show many classic neurofibrillary tangles (NFT) as well as neuritic threads. F) P62 stain of the prefrontal cortex area 10 of DR414, except from the neurofibrillary tangles and neuritic threads containing hyperphosphorylated tau pathology, there is no other immunoreactivity.

**Fig. S5: Prediction of structural changes induced by missense variants in DPP6 with potential effects on glycosylation.**


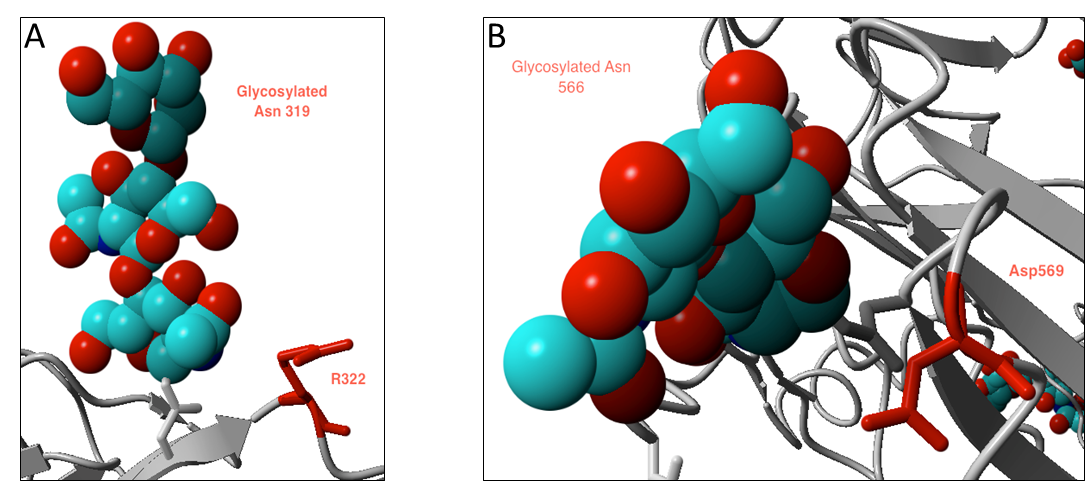


**Fig. S5 legend: A)** Variant p.R322H has a conformational location nearby the glycosylated residue N319 **B)** The variant p.D569N is located next to the glycosylated site N566 and might compete with the latter for the glycosylation.

**Fig. S6: Prediction of structural changes induced by DPP6 missense variant p.K571Q with potential effects on glycosylation.**


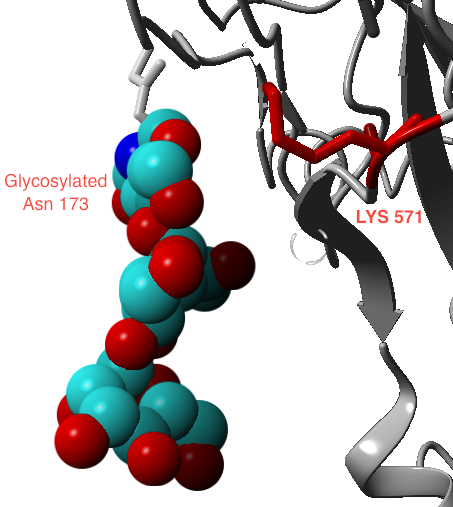

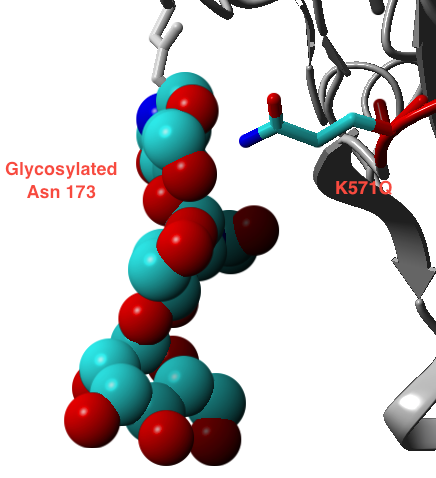


**B**

**A**

**Fig. S6 legend:** A) Wild-type residue and protein conformation. B) The variant p.K571Q can possibly interfere with the glycosylation of the residue N173 for the conformational alteration caused by the amino acid change.

**Fig. S7: *In vitro* protein expression analysis of DPP6-HiBiT constructs**


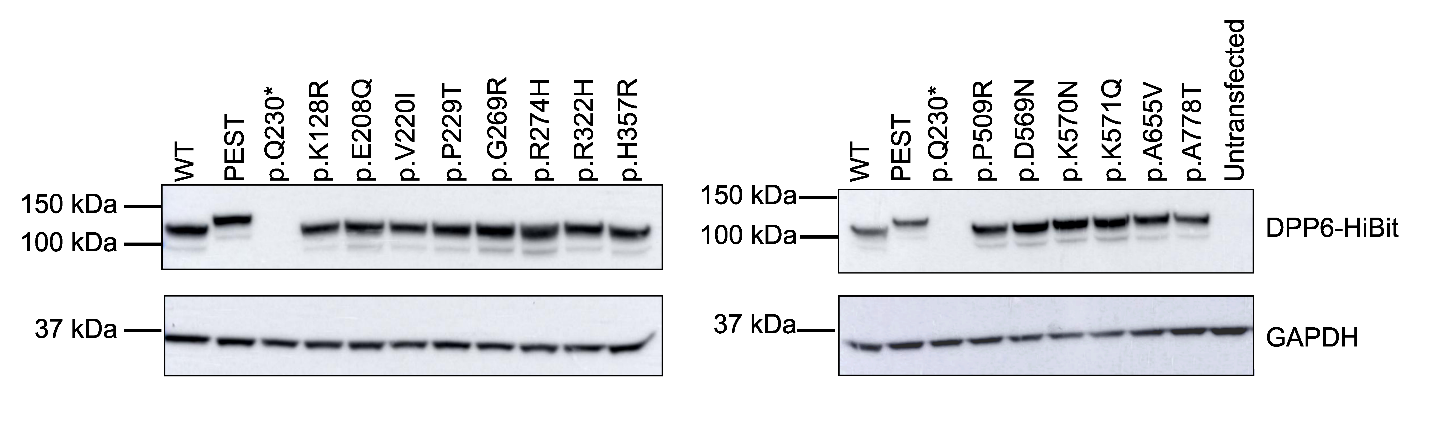


**Fig. S7 legend:** All DPP6 constructs were tested by western blot. The construct with the PEST sequence, shows the expected shift in size. The PTC p.Q230* is used as negative control.

**Immunofluorescence staining**

HEK293T (5x10^5^ cells/well) were transfected in 6-well plates with polyethylenimine (PEI; Polysciences). A total of 4µg of wild-type DPP6 C-terminally fused with HiBit and Lck-GFP were mixed with 5 µL PEI (1 mg/mL) in Opti-MEM (Gibco). Lck-GFP [1] was a gift from Steven Green (Addgene plasmid #61099). Forty-eight hours post-transfection, the cells were fixed with 4 % paraformaldehyde and blocked for 1 hour at room temperature with 10 % goat serum (G9023; Sigma). For the detection, monoclonal mouse anti-DPP6 antibody (1:50; SC-365147 clone [A8]; Santa Cruz Biotechnology) was incubated overnight at 4 °C. After washing with PBS, cells were incubated with Alexa Fluor 594 goat anti-mouse secondary antibody (1:500; Life technologies) for 1 hour at room temperature. DAPI (4’, 6-diamidino-2-phenylindol; 1:10000; D8417; Sigma) was incubated for 10 min. at room temperature after secondary antibody removal with intermittent PBS washes for nuclear staining. Coverslips were mounted into DAKO fluorescence mounting medium (S3023). Immunofluorescence was visualized with a Zeiss LSM 700 confocal microscope using an EC Plan-Neofluar 40x /1.30 Oil objective. Digital images were edited with ImageJ (Fiji).

**Fig. S8: Wild type DPP6-HiBiT localizes on the plasma membrane**

**
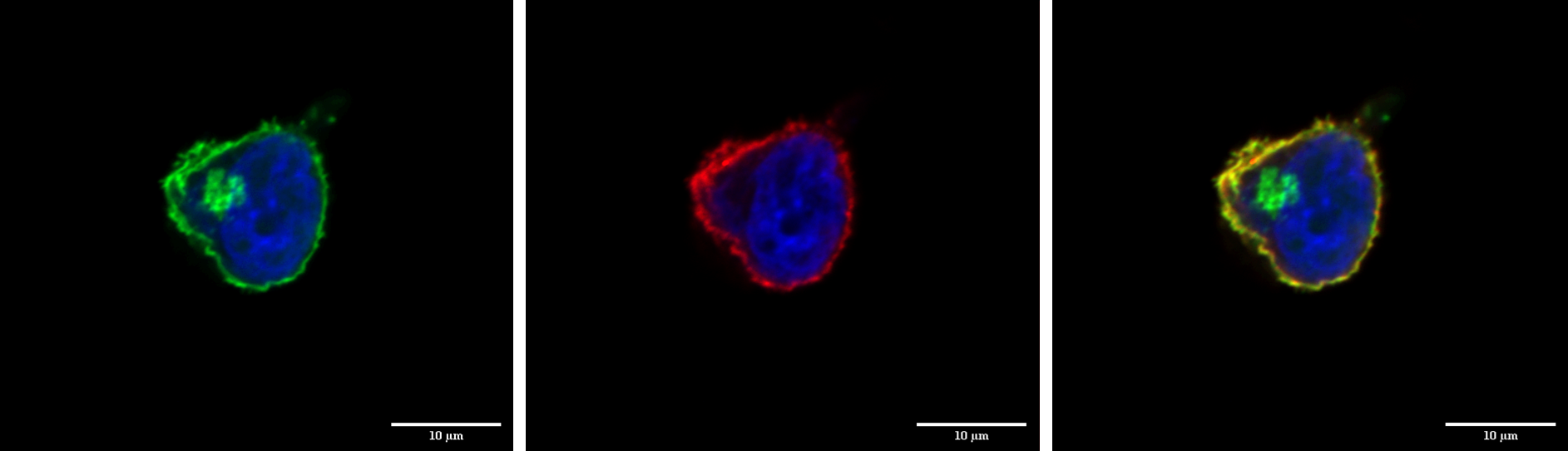
**

A

C

B

**Fig. S8 legend:** Immunocytochemistry of DPP6 with a GFP marker for the plasma membrane was performed on HEK293T cells transiently overexpressing wild-type DPP6 with the C-terminally HiBiT. **A)** LcK-GFP expression as a marker for the plasma membrane (green). **B)** Expression of wild-type DPP6 (red, Alexa Fluor 594). **C)** Co-localization of DPP6 wild-type on the plasma membrane. The scale bar represents 10µm.

**Reference List.**

1. Benediktsson AM, Schachtele SJ, Green SH, Dailey ME (2005) Ballistic labeling and dynamic imaging of astrocytes in organotypic hippocampal slice cultures. J Neurosci Methods 141:41-53. doi:10.1016/j.jneumeth.2004.05.013

2. Bjerke M, Engelborghs S (2018) Cerebrospinal Fluid Biomarkers for Early and Differential Alzheimer's Disease Diagnosis. J Alzheimers Dis 62:1199-1209. doi:10.3233/JAD-170680

3. Cruts M, Gijselinck I, van der Zee J, Engelborghs S, Wils H, Pirici D et al. (2006) Null mutations in progranulin cause ubiquitin-positive frontotemporal dementia linked to chromosome 17q21. Nature 442:920-924

4. Hofman A, Schulte W, Tanja TA, van Duijn CM, Haaxma R, Lameris AJ et al. (1989) History of dementia and Parkinson's disease in 1st-degree relatives of patients with Alzheimer's disease. Neurology 39:1589-1592

5. Niemantsverdriet E, Valckx S, Bjerke M, Engelborghs S (2017) Alzheimer's disease CSF biomarkers: clinical indications and rational use. Acta Neurol Belg 117:591-602. doi:10.1007/s13760-017-0816-5

6. Rademakers R, Cruts M, Dermaut B, Sleegers K, Rosso SM, Van den Broeck M et al. (2002) Tau negative frontal lobe dementia at 17q21: significant finemapping of the candidate region to a 4.8 cM interval. Mol Psychiatry 7:1064-1074

7. van der Zee J, Pirici D, Van Langenhove T, Engelborghs S, Vandenberghe R, Hoffmann M et al. (2009) Clinical heterogeneity in 3 unrelated families linked to VCP p.Arg159His. Neurology 73:626-632. doi:73/8/626 [pii];10.1212/WNL.0b013e3181b389d9 [doi]

8. van Duijn CM, Hendriks L, Farrer LA, Backhovens H, Cruts M, Wehnert A et al. (1994) A population-based study of familial Alzheimer disease: linkage to chromosomes 14, 19, and 21. Am J Hum Genet 55:714-727
